# Effectiveness of a monthly schedule of follow-up for the treatment of uncomplicated severe acute malnutrition in Sokoto, Nigeria: A cluster randomized crossover trial

**DOI:** 10.1371/journal.pmed.1003923

**Published:** 2022-03-01

**Authors:** Matt D. T. Hitchings, Fatou Berthé, Philip Aruna, Ibrahim Shehu, Muhammed Ali Hamza, Siméon Nanama, Chizoba Steve-Edemba, Rebecca F. Grais, Sheila Isanaka

**Affiliations:** 1 Department of Biology, University of Florida, Gainesville, Florida, United States of America; 2 Emerging Pathogens Institute, University of Florida, Gainesville, Florida, United States of America; 3 Epicentre Niger, Niamey, Niger; 4 Médecins Sans Frontières–Operational Center Amsterdam, Amsterdam, the Netherlands; 5 Sokoto State Nutrition Office, Sokoto, Nigeria; 6 UNICEF West and Central Regional Office, Dakar, Senegal; 7 UNICEF Nigeria, Abuja, Nigeria; 8 Epicentre, Paris, France; 9 Departments of Nutrition and Global Health and Population, Harvard T.H. Chan School of Public Health, Boston, Massachusetts, United States of America; The Hospital for Sick Children, CANADA

## Abstract

**Background:**

Community-based management of severe acute malnutrition (SAM) involves weekly or biweekly outpatient clinic visits for clinical surveillance and distribution of therapeutic foods. Distance to outpatient clinics and high opportunity costs for caregivers can represent major barriers to access. Reducing the frequency of outpatient visits while providing training to caregivers to recognize clinical danger signs at home between outpatient visits may increase acceptability, coverage, and public health impact of SAM treatment. We investigated the effectiveness of monthly clinic visits compared to the standard weekly follow-up in the outpatient treatment of uncomplicated SAM in northwestern Nigeria.

**Methods and findings:**

We conducted a cluster randomized crossover trial to test the noninferiority of nutritional recovery in children with uncomplicated SAM receiving monthly follow-up compared to the standard weekly schedule. From January 2018 to November 2019, 3,945 children aged 6 to 59 months were enrolled at 10 health centers (5 assigned to monthly follow-up and 5 assigned to weekly follow-up) in Sokoto, Nigeria. In total, 96% of children (*n* = 1,976 in the monthly follow-up group and 1,802 in the weekly follow-up group) were followed until program discharge, and 91% (*n* = 1,873 in the monthly follow-up group and 1,721 in the weekly follow-up group) were followed to 3 months postdischarge. The mean age at admission was 15.8 months (standard deviation [SD] 7.1), 2,097/3,945 (53.2%) were girls, and the mean midupper arm circumference (MUAC) at admission was 105.8 mm (SD 6.0). In a modified intention-to-treat analysis, the primary outcome of nutritional recovery, defined as having MUAC ≥125 mm on 2 consecutive visits, was analyzed using generalized linear models, with generalized estimating equations to account for clustering. Nutritional recovery was lower in the monthly follow-up group compared to the weekly group (1,036/1,976, 52.4% versus 1,059/1,802, 58.8%; risk difference: −6.8%), and noninferiority was not demonstrated (lower bound of the confidence interval [CI] was −11.5%, lower than the noninferiority margin of 10%). The proportion of children defaulting was lower in the monthly group than in the weekly group (109/1,976, 5.5% versus 151/1,802, 8.4%, *p* = 0.03). Three months postdischarge, children in the monthly group were less likely to relapse compared to those in the weekly group (58/976, 5.9% versus 78/1,005, 7.8%, *p* = 0.03), but cumulative mortality at 3 months postdischarge was higher in the monthly group (159/1,873, 8.5% versus 106/1,721, 6.2%, *p* < 0.001). Study results may depend on context-specific factors including baseline level of care and the clinical status of children presenting to health centers, and, thus, generalizability of these results may be limited.

**Conclusions:**

Where feasible, a weekly schedule of clinic visits should be preferred to maintain effectiveness of SAM treatment. Where geographic coverage of programs is low or frequent travel to outpatient clinics is difficult or impossible, a monthly schedule of visits may provide an alternative model to deliver treatment to those in need. Modifications to the outpatient follow-up schedule, for example, weekly clinic visits until initial weight gain has been achieved followed by monthly visits, could increase the effectiveness of the model and add flexibility for program delivery.

**Trial registration:**

ClinicalTrials.gov NCT03140904.

## Abstract 2

Matt D.T. Hitchings and colleagues investigate the effectiveness of a monthly schedule of follow-up for the management of uncomplicated severe acute malnutrition in children aged 6-59 months in Nigeria.

## Author summary

**Why was this study done?:**

- Severe acute malnutrition (SAM) affects at least 13.6 million children under 5 worldwide each year and is associated with significant increase in mortality and morbidity.
- Despite improvements in the management of SAM, coverage of treatment programs remains low. Distance to outpatient clinics and high opportunity costs for caregivers represent major barriers to access.
- New models are needed to increase coverage and access to lifesaving treatment for SAM.

**What did the researchers do and find?:**

- We conducted a cluster randomized crossover trial to assess the effectiveness of a monthly follow-up schedule compared to a weekly schedule in terms of nutritional recovery and secondary outcomes including nonresponse, default, hospitalization, and death, and weight gain, length of stay, and relapse among recovered children. Study outcomes were assessed at the end of treatment and at 3 months postdischarge.
- Noninferiority of the monthly follow-up group in terms of nutritional recovery was not demonstrated. However, we found lower rates of default with monthly follow-up implying greater acceptability among caregivers. There was no statistical difference in weight velocity between groups at 4 and 8 weeks, but time to recovery was longer with monthly follow-up and relapse at 3 months postdischarge was less likely to occur in the monthly group. Finally, the mortality risk between admission and 3 months postdischarge was significantly higher in the monthly follow-up group.

**What do these findings mean?:**

- Where possible, a weekly schedule of clinic visits should be preferred for treatment of SAM.
- Although this study did not demonstrate noninferiority of a monthly schedule of follow-up for nutritional recovery, monthly follow-up may be considered in specific settings where more frequent follow-up is infeasible.

## Introduction

Severe acute malnutrition (SAM) is a life-threatening condition that affects at least 13.6 million children under 5 each year worldwide [1]. SAM is associated with a greatly increased risk of death [2–4] and contributes to nearly half of all childhood deaths worldwide [5]. Community-based management of acute malnutrition (CMAM), endorsed by the United Nations in 2007 [6], has proven effective, but coverage remains low. It is estimated that, globally, only 37% of children in need receive treatment [1,7]. Distance to outpatient clinics and high opportunity costs for caregivers represent major barriers to access [8,9].

New models are needed to increase coverage and access to lifesaving treatment for SAM. One alternative model may employ a reduced schedule of outpatient follow-up, paired with monthly distribution of therapeutic foods and caregiver education and support for at-home clinical surveillance in between scheduled clinic visits. A monthly schedule of follow-up would reduce the burden on caregivers and service providers that is associated with weekly clinic visits and would support caregiver engagement to monitor the clinical and anthropometric status of children from home between scheduled clinic visits.

Here, we report the results of a cluster randomized crossover trial comparing the effectiveness of a monthly schedule follow-up to the standard weekly schedule follow-up in northwestern Nigeria. The objective of the trial was to provide evidence to develop flexible programming to increase the coverage of SAM treatment where standard weekly follow-up is not feasible or acceptable.

## Methods

We conducted a cluster randomized crossover trial of a monthly schedule of clinic visit follow-up in the treatment of uncomplicated SAM among children aged 6 to 59 months with a noninferiority design (ClinicalTrials.gov ID: NCT03140904). The study protocol and the STrengthening the Reporting of OBservational studies in Epidemiology (STROBE) Checklist are provided as S1 Protocol and S1 STROBE Checklist.

### Study setting

The study was conducted in 10 outpatient clinics in the Binji and Wamako local government areas (LGA) in Sokoto state in northwestern Nigeria. This setting is a rural area largely representative of the Sahel region with endemic malnutrition and seasonal peaks of acute malnutrition during the lean season prior to harvest. While national SAM prevalence in Nigeria is 2% [10], northwestern Nigeria experiences the highest rates of malnutrition in the country, with reported SAM prevalence of 7.9% in children <5 years old in Sokoto state in 2018 [10]. CMAM was introduced in northern Nigeria in 2009, and by 2014, achieved coverage of 37% of children eligible for care [11]. The program includes clinic-based care provided by trained health care workers. SAM treatment was provided free of charge in all 10 study sites by the Sokoto State Ministry of Health (SMOH), with support from UNICEF.

### Study population

The study population included children newly admitted for the treatment of uncomplicated SAM at 1 of 10 outpatient clinics between January 2018 and November 2019. Eligibility criteria for treatment of uncomplicated SAM according to local program criteria were minimum weight of 3.5 kg, midupper arm circumference (MUAC) <115 mm and/or grades 1 and 2 edema, absence of current illness requiring inpatient care, and age 6 to 59 months. Additional inclusion criteria were residence in the catchment area of one of the study clinics and written informed consent of the parent or legal guardian. Cases of relapse, where the child was previously successfully treated, discharged as cured and returned with a new episode of acute malnutrition within 2 months of discharge, were eligible for admission, as were those transferred from inpatient care to outpatient care to continue treatment. Returned defaulters who were absent for 3 consecutive visits before recovery and returned to continue treatment and children transferred from another outpatient site were not eligible. Other exclusion criteria for the study included reported history of allergy to peanuts and any other condition which, in the judgment of the field investigator, would interfere with or serve as a contraindication to protocol adherence or the ability to give informed consent.

### Study intervention

The study compared 2 schedules of follow-up:

- standard weekly visits at the outpatient clinic until program discharge and
- monthly visits at the outpatient clinic with caregiver support for home-based clinical surveillance, with visits scheduled at weeks 4, 8, 10, and 12 until program discharge.

The proposed monthly schedule of follow-up involved an extension of the period between which the child received a physical assessment by a trained health worker from 1 week to 1 month. During this time, caregivers were asked to monitor the clinical and anthropometric status of the child at home and return to the outpatient clinic for medical attention upon development of any clinical sign of concern (e.g., diarrhea, vomiting, fever, lethargy, lack of appetite, edema, cough, difficulty breathing, or convulsions). To support caregivers in these tasks, culturally appropriate educational tools and messages, including materials for facility-based instruction (e.g., posters and pictorial flip charts), as well as for home-based use (e.g., pocket pictorial cards), were developed with Médecins Sans Frontières France and the Laboratoire d’Etudes et de Recherche sur les Dynamiques Sociales et le Développement Local (LASDEL) in 2014. The tools covered the key topics of (a) home-based MUAC measurement; (b) clinical surveillance of key danger signs or symptoms; and (c) appropriate storage and use of the monthly therapeutic ration. The feasibility and acceptability of these tools were tested in a pilot study in Madarounfa, Niger [12,13]. Caregiver understanding of clinical danger signs increased following training and agreement between MUAC measurements was high between nurses and caregivers.

All children admitted for outpatient treatment at a participating study clinic received the same schedule of follow-up (e.g., weekly or monthly clinic visits). Due to lower than expected enrollment in the first 11 months of the study, a 12-month extension of the enrollment period, together with a crossover design to increase study power and efficiency [14], was implemented. On December 17, 2018, the crossover was executed in the field: Outpatient clinics that had been administering monthly visits began to administer weekly visits to all newly admitted children and vice versa. Intervention assignment among children already under follow-up did not change at the time of crossover, and children were analyzed as the group their sites were randomized to at the time of their enrolment. As crossover occurred at the site level, staff ensured children already enrolled completed treatment until discharge under their site’s initial treatment and administered the new crossover treatment only to newly enrolled children. There was no washout period at the site level.

### Study follow-up and procedures

At each follow-up visit at the health clinic, a physical exam and anthropometric surveillance were performed, and a therapeutic ration of ready-to-use therapeutic food (RUTF) was distributed. Study staff carried out anthropometric measurements with the use of standardized methods and calibrated instruments [15]. Child height (recumbent length if <85 cm) was measured to the nearest 0.1 cm using a wooden measurement board. Weight was measured to the nearest 0.1 kg using electronic seca scales. Standard medical care was provided as per the national protocol for the treatment of acute malnutrition [16].

As per study protocol, all children received an unannounced home visit within 2 weeks of admission by community health workers to assess the safety of the program and a 3-month postdischarge clinic visit to assess longer-term outcomes. At the home safety visit, study staff assessed incident morbidities, measured MUAC, and counted the number of used RUTF sachets in the household as a proxy to assess appropriate use of the RUTF rations early in treatment. Children with clinical danger signs identified at the home safety visit were referred to the health center for care, and fees for transportation to the hospital, medicine, and testing during eventual inpatient care were taken in charge by the study.

To measure RUTF adherence, caregivers were instructed to return to each scheduled clinic visit with used and unused RUTF sachets distributed at the previous visit. A child was defined as compliant if, at the end of treatment, their caregiver returned used at least 80% of sachets provided before the final clinic visit of treatment.

### Blinding and randomization

The unit of randomization was the SMOH-supported outpatient clinic. The 10 health clinics in Binji and Wamako LGAs were stratified by size of admission (6 sites with ≥500 admissions per year and 4 sites with <500 admissions per year), and centers within each stratum were randomized in a 1:1 ratio to one of the 2 schedules of follow-up. Randomization assignment was made by lottery, in which a local representative selected the name of one of the 2 interventions from an opaque plastic jar. Due to the nature of the intervention, neither the investigators nor participants were blinded.

### Outcomes

The primary effectiveness outcome was nutritional recovery, defined in both groups as being free from medical complications, having MUAC ≥125 mm on 2 consecutive clinic visits, and no edema.

Secondary effectiveness outcomes assessed at program discharge were nonresponse (defined as not meeting the definition of nutritional recovery by 12 weeks from admission); default (defined as 3 missed scheduled clinic visits in the weekly follow-up group and 1 missed scheduled clinic visit in the monthly follow-up group); hospitalization (defined as all-cause, due to weight/edema changes, and/or due to clinical complication, and not including events that ended in death); death from any cause; and among recovered children, weight gain (g/kg/day) at week 4 and week 8 from admission and at program discharge and length of stay from admission to program discharge.

At each scheduled clinic visit, incident morbidities assessed by study staff or caregiver report included edema, diarrhea, vomiting, fever, cough, lack of appetite, high temperature, tachypnea, dehydration, pallor, and superficial skin infection at the clinic visit or in the previous week. To assess the early safety of the program at the 2-week home visit, morbidities and change in MUAC (mm/day) from the admission visit to the home visit were recorded. In addition, the RUTF deviance was calculated as the difference between the number of used RUTF sachets observed at home compared to the number expected based on the time since admission and the child’s prescribed dose, as described previously [12]. At 3 months postdischarge, longer-term outcomes assessed were all-cause hospitalization, all-cause cumulative mortality, and relapse after treatment recovery (defined as a maternal report of admission to any therapeutic feeding program within 3 months of discharge from the index admission or MUAC <115 mm or edema observed at the time of the postdischarge visit).

### Sample size

Given an observed probability of nutritional recovery in the weekly follow-up group of 0.55, intracluster correlation of 0.01, between-cluster between-period correlation of 0, significance level of 0.05 using the 1-sided Score test, and allowing for 10% loss to follow-up, a sample size of 175 children per outpatient clinic in each period (1,750 per group) was sufficient to achieve >90% power to detect a noninferiority margin difference between the group proportions of −0.10 [17]. The noninferiority margin of 10% was chosen to represent a difference in recovery with programmatic and policy importance within this context.

### Statistical analysis

The analysis of the primary outcome, nutritional recovery, was a noninferiority analysis. If the lower bound of the observed risk difference confidence interval (CI) was greater than the noninferiority margin of −0.10, there would be evidence that monthly follow-up was noninferior to weekly follow-up. To estimate risk difference for nutritional recovery, we used a binomial regression with an identity link. For each of the other program outcomes, morbidity at the home safety visit, RUTF adherence, and all postdischarge outcomes, we used log-binomial regression to estimate relative risk and associated 95% CIs comparing intervention groups. Weight gain and length of stay among recovered children, as well as change in MUAC observed at the safety visit, were analyzed using linear regression. We analyzed the incidence of morbidities at clinic visits using Poisson regression, with the number of visits as an offset term. For every analysis, we used generalized estimating equations with clustering by site within each crossover period [18], choosing a full model accounting for clustering by site between crossover periods where appropriate [19]. We defined the lean season as the period of increased admission before the harvest period (July to October) and included an interaction between intervention group and season to assess differences in program effectiveness and RUTF adherence by season. Finally, we assessed differences in RUTF adherence by whether there were ±2 children under 5 years living in the household by including an interaction term. All analyses were performed on the modified intention-to-treat basis, which excluded individuals with protocol violations (*n* = 72) and those who withdrew consent during the study (*n* = 99). Data entry was performed using EpiInfo version 7.2.2.2, and sample size calculations and analyses were performed using SAS version 9.3 (SAS Institute, Cary, North Carolina, United States of America) and R, version 4.0.2.

### Ethical considerations

The study was approved by the research ethics committee of Harvard T.H. Chan School of Public Health and the SMOH, Nigeria and was conducted in accordance with the Declaration of Helsinki. An independent data and safety monitoring board reviewed study progress and safety events. Caregivers provided written informed consent before admission and were made aware of their ability to withdraw from the study at any time.

## Results

**Fig 1:**
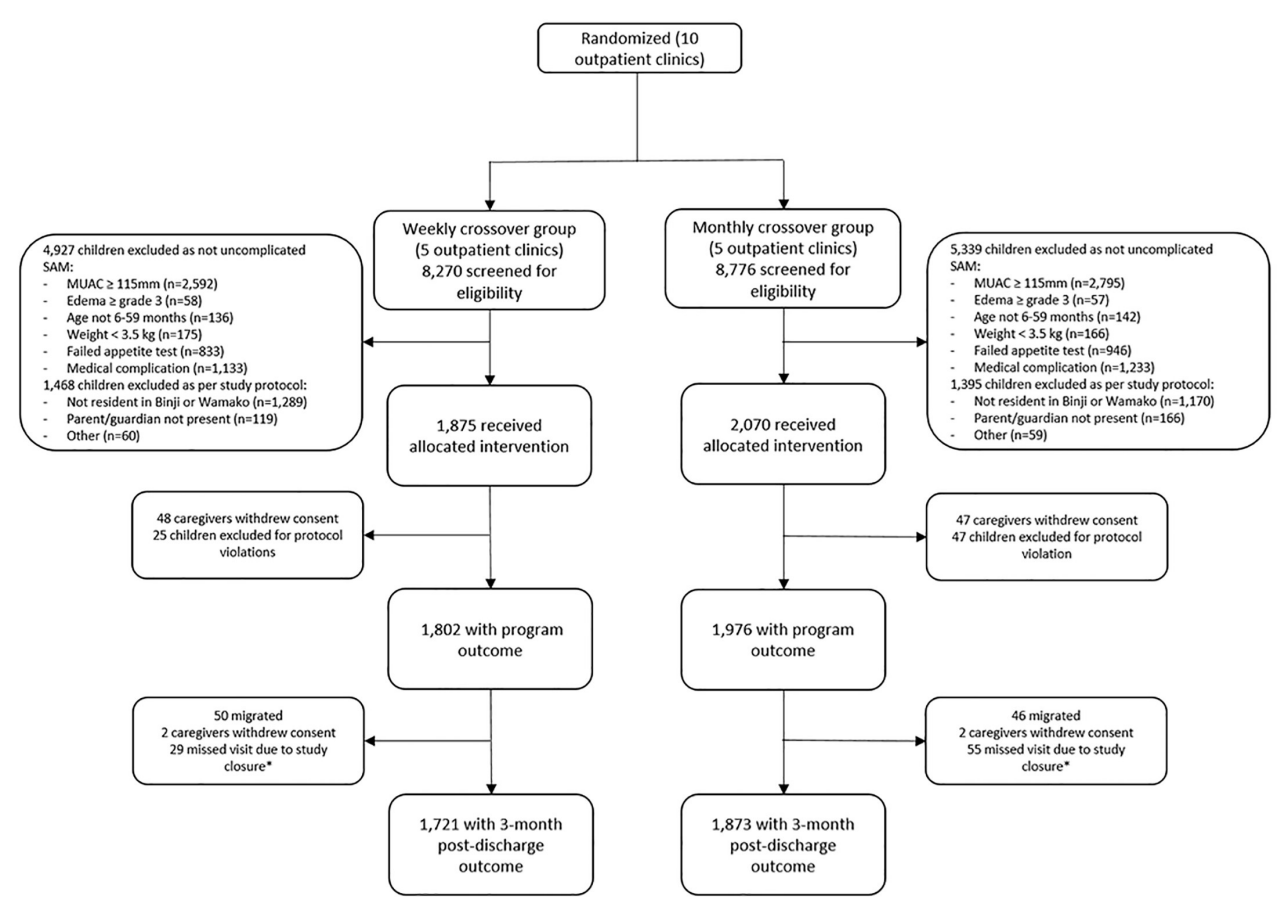
CONSORT flowchart of enrollment, intervention assignment, and analysis of program and postdischarge outcomes. *Field activities were suspended in April 2020 due to COVID-19–related precautions. This early closure was 3 weeks in advance of the planned closure date. At the time, follow-up for the primary outcome was complete. COVID-19, Coronavirus Disease 2019; MUAC, midupper arm circumference; SAM, severe acute malnutrition.

Between January 2018 and November 2019, 6,780 children presented to the 10 outpatient clinics with uncomplicated SAM (Fig 1). After screening, 3,945 children were eligible for the study and were enrolled in one of the 2 intervention groups as per the clinic random assignment. A total of 3,778 children (1,802 in the weekly follow-up group and 1,976 in the monthly follow-up group) were included in the analysis to program discharge, and 3,594 children (1,721 in the weekly follow-up group and 1,873 in the monthly follow-up group) were followed to 3 months postdischarge.

Table 1 describes the characteristics of the study participants and their households measured at admission. The mean age at admission was 15.8 months (standard deviation [SD] 7.1), 53.2% were girls, and the mean MUAC at admission was 105.8 mm (SD 6.0). The study population was characterized by low levels of maternal education (5.7% with any education) and large household sizes (mean 6.7 children per household). Children were enrolled with uncomplicated SAM with few clinical signs as per study protocol. The most prevalent clinical signs at admission were dehydration and cough, but with little to no difference between groups.

**Table 1:** Baseline characteristics of the study population by intervention group.

Characteristic	Total (*n* = 3,945)	Weekly (*n* = 1,875)	Monthly (*n* = 2,070)
Sociodemographic characteristics^1^			
Child’s age, months	15.8 (7.1)	15.7 (7.1)	15.8 (7.2)
Female sex	2,097 (53.2%)	962 (51.3%)	1,135 (54.8%)
Mother’s age, years	26.2 (6.5)	26.4 (6.8)	26.0 (6.2)
Distance to clinic by foot (minutes)	57.3 (39.0)	55.3 (35.4)	59.2 (42.0)
Mother’s education (any)	225 (5.7%)	113 (6.1%)	112 (5.4%)
Mother’s number of live births	3.5 (2.0)	3.5 (2.0)	3.5 (2.0)
Anthropometric data			
Child’s weight, kg	5.8 (1.1)	5.8 (1.1)	5.8 (1.1)
Child’s weight-for-height z-score^2^	−3.8 (1.2)	−3.8 (1.2)	−3.7 (1.1)
Child’s MUAC, mm	105.8 (6.0)	105.7 (6.1)	105.9 (6.0)
Child’s height, cm	68.7 (6.4)	68.6 (6.4)	68.7 (6.5)
Medical and feeding history at admission (maternal report)			
Diarrhea	42 (1.1%)	20 (1.1%)	22 (1.1%)
Vomiting	26 (0.7%)	13 (0.7%)	13 (0.6%)
Cough	76 (1.9%)	31 (1.7%)	45 (2.2%)
Edema	27 (0.7%)	13 (0.7%)	14 (0.7%)
Poor or no appetite	21 (0.5%)	4 (0.2%)	17 (0.8%)
Physical examination and diagnostic tests by program staff			
Thirsty	1,812 (46.0%)	860 (45.9%)	952 (46.0%)
Physical signs of dehydration	124 (3.1%)	32 (1.7%)	92 (4.5%)

^1^Continuous variables are displayed as mean (SD). Binary variables are displayed as *n* (%).

^2^WHO Child Growth Standards [31].

MUAC, midupper arm circumference; SD, standard deviation.

The average number of scheduled clinic visits per child was 8.5 (SD 3.1) in the weekly follow-up group and 4.2 (SD 1.3) in the monthly follow-up group. The percentage of children achieving nutritional recovery in the monthly follow-up group was lower than in the weekly follow-up group (52.4% versus 58.8%; risk difference: −6.8%). The lower bound of the 1-sided CI was −11.5%, therefore lower than the 10% noninferiority margin. Recovery varied by season, with lower recovery (and higher nonresponse) among children admitted in the monthly follow-up group during the lean season but no statistical difference between groups outside of the lean season (p for interaction = 0.05, Table A in S1 Text).

There was no statistical difference in hospitalization overall by group; however, the risk of hospitalization due to clinical complications was lower (risk ratio (RR) = 0.60, 95% CI: 0.47 to 0.77, *p* < 0.001) and risk of hospitalization due to weight loss/edema was higher in the monthly follow-up group (RR = 2.60, 95% CI: 1.86 to 3.63, *p* < 0.001). The risk of nonresponse was significantly higher in the monthly follow-up group compared to the weekly follow-up group (36.8% versus 29.0%; RR 1.28, 95% CI: 1.07 to 1.53, *p* = 0.01, Table 2). The most common causes of death were gastroenteritis (49%), malaria (17%), and lower respiratory tract infection (18%) (Table B in S1 Text); there was no significant difference between groups in the risk of death at program discharge (RR 1.38, 95% CI: 0.98 to 1.95, *p* = 0.06).

**Table 2:** Effect of monthly schedule of follow-up compared to standard weekly schedule of follow-up on primary and secondary outcomes assessed at program discharge.

	Weekly follow-up *n* (%)	Monthly follow-up *n* (%)	Risk difference (95% 1-sided CI lower bound)^1^	*p*-Value
*N*	1,802	1,976		
Nutritional recovery	1,059 (58.8%)	1,036 (52.4%)	−6.8% (−11.5%)	-
			RR (95% CI)^2^	
Hospitalization	277 (15.4%)	267 (13.5%)	0.88 (0.65, 1.19)	0.41
Due to weight loss or edema	52 (2.9%)	151 (7.6%)	2.60 (1.86, 3.63)	<0.001
Due to clinical complications	224 (12.4%)	150 (7.6%)	0.60 (0.47, 0.77)	<0.001
Nonresponse	522 (29.0%)	727 (36.8%)	1.28 (1.07, 1.53)	0.01
Default	151 (8.4%)	109 (5.5%)	0.66 (0.45, 0.97)	0.03
Death	70 (3.9%)	104 (5.3%)	1.38 (0.98, 1.95)	0.06

^1^Risk difference and 1-sided upper 95% CI from generalized estimating equations with an exchangeable correlation structure, a binomial distribution, and an identity link.

^2^RR and 95% CI from generalized estimating equations with an exchangeable correlation structure, a binomial distribution, and a log link.

CI, confidence interval; RR, risk ratio.

Among children who recovered, the length of stay was significantly longer in the monthly follow-up group (67.4 days (SD 11.0) versus 51.2 days (SD 18.6), *p* < 0.001). There was no significant difference observed in weight change among recovered children between groups at 4 or 8 weeks from admission (6.4 g/kg/day overall at 4 weeks, *p* = 0.70 and 5.0 g/kg/day overall at 8 weeks, *p* = 0.40).

The study protocol included an unannounced home visit 2 weeks after admission to monitor the early safety of the intervention. The prevalence of morbidities and change in MUAC from admission observed at the home visit was similar between groups (Table 3). However, mean RUTF deviance (e.g., observed minus expected count of used RUTF sachets) was significantly higher in the monthly follow-up group (3.3 sachets (SD 9.8) versus 1.6 sachets (SD 3.7), *p* = 0.03), suggesting better early adherence to the prescribed dose in the weekly follow-up group.

**Table 3:** Effect of monthly schedule of follow-up compared to standard weekly schedule of follow-up on clinical signs and anthropometric status at the 2-week home safety visit.

	Weekly follow-up *n* (%)	Monthly follow-up *n* (%)	RR (95% CI)^1^	*p*-Value
*N*	1,778	1,947		
Diarrhea	299 (16.8%)	462 (23.7%)	1.29 (0.89, 1.86)	0.18
Edema	8 (0.5%)	9 (0.5%)	0.95 (0.45, 2.02)	0.90
Vomiting	114 (6.4%)	138 (7.1%)	1.26 (0.66, 2.4)	0.49
Fever	317 (17.8%)	380 (19.5%)	1.12 (0.79, 1.57)	0.53
Cough	227 (12.8%)	254 (13.1%)	1.08 (0.64, 1.82)	0.78
Respiratory distress	47 (2.7%)	35 (1.8%)	0.85 (0.38, 1.9)	0.69
Lost appetite	105 (5.9%)	129 (6.6%)	1.3 (0.54, 3.15)	0.56
Convulsions	6 (0.3%)	5 (0.3%)	0.71 (0.19, 2.68)	0.62
Lethargy	42 (2.4%)	77 (4.0%)	1.7 (0.93, 3.11)	0.08
	Weekly follow-up Mean (SD)	Monthly follow-up Mean (SD)	Mean difference (95% CI)^2^	
Change in MUAC per day	0.5 (0.6)	0.5 (0.6)	0.0 (−0.1, 0.5)	0.70

^1^RR and 95% CI from generalized estimating equations with an exchangeable correlation structure, a binomial distribution, and a log link.

^2^Mean difference and 95% CI from generalized estimating equations with an exchangeable correlation structure, with a normal distribution for the outcome.

CI, confidence interval; MUAC, midupper arm circumference; RR, risk ratio; SD, standard deviation.

Over the duration of treatment, there was no consistent pattern in differences in morbidity by group: Children in the monthly follow-up group experienced significantly higher risk of lack of appetite and dehydration, but lower risk of diarrhea, superficial skin infection, vomiting, fever, and cough over time (Table 4).

**Table 4:** Effect of monthly schedule of follow-up compared to standard weekly schedule of follow-up on clinical signs reported or observed at scheduled clinic visits during treatment.

	Weekly follow-up *n* (%)	Monthly follow-up *n* (%)	Rate ratio (95% CI)^1^	*p*-Value
*N*, children	1,802	1,976		
*N*, follow-up visits	17,184	8,762		
Diarrhea	560 (3.3%)	226 (2.6%)	0.81 (0.69, 0.95)	0.01
High temperature (>38.5°C)	42 (0.2%)	22 (0.3%)	1.02 (0.59, 1.77)	0.94
Tachypnea	21 (0.1%)	10 (0.1%)	1.04 (0.49, 2.22)	0.92
Pallor/anemia	22 (0.1%)	7 (0.1%)	0.60 (0.22, 1.68)	0.33
Edema	60 (0.4%)	20 (0.2%)	0.68 (0.37, 1.25)	0.22
Lack of appetite	142 (0.8%)	101 (1.2%)	1.40 (1.04, 1.89)	0.03
Dehydration	133 (0.8%)	157 (1.8%)	2.53 (1.84, 3.49)	<0.001
Superficial skin infection	90 (0.6%)	22 (0.3%)	0.42 (0.26, 0.7)	<0.001
Vomiting	262 (1.5%)	86 (1.0%)	0.65 (0.5, 0.83)	<0.001
Fever in the last week by maternal report	880 (5.1%)	289 (3.3%)	0.65 (0.55, 0.73)	<0.001
Cough	729 (4.3%)	215 (2.5%)	0.56 (0.47, 0.67)	<0.001

^1^Rate ratio and 95% CI from generalized estimating equations with an exchangeable correlation structure and a Poisson distribution.

CI, confidence interval.

Longer-term impacts of the intervention were assessed at a 3-month postdischarge visit. Among children who recovered, the proportion who relapsed within 3 months postdischarge was significantly lower in the monthly follow-up group (RR = 0.73, 95% CI 0.56 to 0.96, *p* = 0.03). However, the cumulative proportion of children who died between admission and the 3 months postdischarge visit was significantly higher among the monthly follow-up group (RR = 1.39 95% CI 1.15 to 1.68, *p* < 0.001, Table 5).

**Table 5:** Effect of monthly schedule of follow-up compared to standard weekly schedule of follow-up on outcomes up to 3 months postprogram discharge.

	Weekly follow-up *n* (%)	Monthly follow-up *n* (%)	RR (95% CI)^1^	*p*-Value
*N*	1,721	1,873		
Death	106 (6.2%)	159 (8.5%)	1.39 (1.15, 1.68)	<0.001
Hospitalization	299 (17.4%)	292 (15.6%)	0.90 (0.67, 1.20)	0.46
*N*, recovered	1,005	976		
Relapse^2^	78 (7.8%)	58 (5.9%)	0.73 (0.56, 0.96)	0.03

^1^RR and 95% CI from generalized estimating equations with an exchangeable correlation structure, a binomial distribution, and a log link.

^2^Relapse defined as program admission since index discharge by maternal report or MUAC <115 mm or edema measured at 3-month postdischarge visit.

CI, confidence interval; MUAC, midupper arm circumference; RR, risk ratio.

Adherence to RUTF use, measured in this study by returned sachet count, was high in both groups: Overall, 99% of caregivers returned ≥80% of distributed RUTF sachets during treatment. However, the monthly follow-up group was slightly less likely to be adherent across all of follow-up (98.0% compliant versus 99.6% compliant; RR = 0.99, 95% CI 0.98 to 1.00, *p* = 0.05). The difference in RUTF adherence did not vary between groups by season or by household size.

## Discussion

This trial investigated the effectiveness of a monthly schedule of follow-up in the outpatient treatment of uncomplicated SAM. Noninferiority of the monthly follow-up group in terms of nutritional recovery was not demonstrated. However, we found lower rates of default with monthly follow-up implying greater acceptability among caregivers. There was no statistical difference in weight velocity between groups at 4 and 8 weeks, but time to recovery was longer with monthly follow-up, and relapse at 3 months postdischarge was less likely to occur in the monthly group. While there has been limited evaluation of reduced schedules of follow-up, including from a pilot study in Niger [12,13], this study represents the first rigorous evaluation of the effectiveness of such a model conducted for outcomes of clinical and programmatic interest at larger scale.

Hospitalization represents an important adverse outcome in the treatment of any child. While we found no statistical difference between the risk of hospitalization overall or of death during treatment, we noted decreased hospitalization due to clinical complications and elevated mortality in the monthly follow-up group. It is possible that children in the monthly follow-up group may not have presented to the hospital when clinical care was required, thus suffering from higher mortality at home. In the study setting, the majority of hospitalizations were referred from the clinic rather than from self-referral, and children in the weekly follow-up group were more likely to be referred during a clinic visit than children in the monthly follow-up group. In settings where inpatient care-seeking behavior is limited, frequent follow-up with health staff may be crucial in reducing the risk of adverse outcomes such as hospitalization. The increased risk of hospitalization due to weight loss/edema in the monthly follow-up group may be supported by the finding of greater RUTF deviance at the home safety visit. Adverse risks of hospitalization due to weight loss/edema may be mitigated through more community-based support for appropriate RUTF use at home.

The proportion of children defaulting from the program was lower in the monthly follow-up group. A number of common barriers to access SAM treatment have been identified [8,9], including high opportunity costs to caregivers due to long and frequent visits to treatment centers and distance to the health center. High acceptability of a monthly schedule could be a key component to improving coverage and public health impact of SAM treatment programs. In this setting, the average time taken to reach the outpatient clinic was over 60 minutes, and so by reducing the frequency of such trips and the burden on the caregivers, the monthly follow-up schedule reduced defaulting and appeared to have greater acceptability.

The education sessions delivered to caregivers were a key component of the monthly follow-up intervention. Caregiver education led to significant and sustained improvement in knowledge of home-based MUAC measurement and key clinical danger signs or symptoms (analysis forthcoming). However, the development of materials and training of study staff can represent a significant investment of time before use. We emphasize the importance of the educational materials being context specific to align with the epidemiological profile and cultural practices of the setting.

The overall risk of nutritional recovery (55.5%) in this setting was low compared to international standards [20], although observed weight gain velocity among recovered children was similar to that reported in other published studies of community-based SAM management [21–24]. Low recovery in both groups may be attributed to insufficient health systems and access to medical care. Although health centers provided routine medicines when available and the study reimbursed caregivers for transportation, medicines, and testing during inpatient care, our findings suggest that the strong provision and uptake of medical services may be essential alongside the simple provision of therapeutic foods in order to achieve high recovery. Low recovery in both groups may have also been associated with suboptimal usage of RUTF at home. The study observed high RUTF adherence in both groups as recorded by RUTF sachets returned to scheduled clinic visits, but we were unable to directly ascertain whether the index child had received the correct dose of RUTF at home throughout treatment. We found recovery to be lower among children admitted in the monthly follow-up group during the lean season, which could be due to increased intra- and interhousehold sharing of RUTF in times of greater food insecurity.

The global nutrition community is motivated to identify alternative models that can improve the coverage, cost, and performance of SAM management [25–28]. Although this study did not demonstrate noninferiority of a monthly schedule of follow-up for nutritional recovery, monthly follow-up may be considered in specific settings where more frequent follow-up is infeasible. Where regular movement to an outpatient clinic is difficult or impossible due to conflict, Coronavirus Disease 2019 (COVID-19) restrictions, or long travel times, and in particular where the alternative is no treatment, monthly follow-up could provide an acceptable model to increase program coverage and save lives. Our results suggest that modifications could be considered to improve the effectiveness of the monthly follow-up schedule, for example, by maintaining weekly follow-up early in treatment until initial weight gain has been achieved or among children with certain risk factors such as mild clinical complications, which may help identify important clinical danger signs early at health centers and reduce adverse events. Support for timely care-seeking behavior and appropriate RUTF use at home should be considered critical components of this model if applied elsewhere. As the increased length of stay associated with the reduced follow-up schedule can have implications for program costs, cost-effectiveness analysis may also be helpful to identify specific settings where this model could be acceptable.

The study has some limitations. The generalizability of these results may be limited by the context in which the study was conducted. Care was available at outpatient clinics that were operational 1 day per week, which may have led to constrained baseline care-seeking behavior. We found the proportion of children initially presenting to outpatient clinics with clinical complications was high (Fig 1), indicating that presentation to health clinics was frequently sought late in the SAM episode. The frequency of self-referred hospitalization was also low in the study even after educational sessions on clinical danger signs and reimbursement for usual expenses associated with inpatient care, which may have reduced the effectiveness of the monthly follow-up schedule. Effectiveness and acceptability may differ in populations with a lower burden of clinical complications and different care-seeking behavior. As per the study protocol, the definition of nutritional recovery was more demanding in the monthly arm than in the weekly arm, possibly underestimating the recovery in the monthly arm. While low recovery rate affected the initial power of the study, the crossover design and extension of the enrollment period sought to correct this and adequate power (>90%) to assess the primary outcome was achieved by study closure.

The design and rigorous conduct of this study lends strength to our findings. First, a high proportion (96%) of children were followed to program discharge, reducing the risk of selection bias due to differential loss to follow-up. Second, the 2-week home visit allowed us to report on the early safety of the intervention in the period immediately following admission and refer children with incident clinical complications to care, thus reducing mortality in both groups. Third, longer-term outcomes following treatment for SAM have not been widely reported, although it has been observed that significant increased mortality can occur after recovery [29,30]. Our study was able to add important evidence to longer-term intervention effects, importantly showing lower relapse but higher overall mortality in the monthly follow-up group 3 months postdischarge.

In conclusion, this study demonstrated that a monthly schedule of follow-up for the outpatient treatment of uncomplicated SAM was not noninferior to a weekly schedule of follow-up. In our study setting of northwestern Nigeria, and generally where weekly follow-up programs are recommended and feasible, weekly follow-up schedules should be preferred to prevent adverse events through close clinical monitoring by health staff. Monthly follow-up with support for at-home monitoring of children’s clinical and anthropometric status may provide an alternative model in contexts in which frequent access to health centers is limited. Adaptations to this reduced monthly schedule of follow-up, for example, with weekly follow-up only until initial weight gain has been achieved, could be considered to reduce overall patient burden while maintaining safety.

## Supporting information

S1 STROBE Checklist STROBE Checklist. STROBE, STrengthening the Reporting of OBservational studies in Epidemiology.
(DOCX)

S1 Protocol Study protocol. (DOCX)

S1 Text Supporting information Tables A and B. (DOCX)

## Abbreviations

CI: confidence interval
CMAM: community-based management of acute malnutrition
COVID-19: Coronavirus Disease 2019
LASDEL: Laboratoire d’Etudes et de Recherche sur les Dynamiques Sociales et le Développement Local
LGA: local government area
MUAC: midupper arm circumference
RUTF: ready-to-use therapeutic food
SAM: severe acute malnutrition
SD: standard deviation
SMOH: Sokoto State Ministry of Health
STROBE: STrengthening the Reporting of OBservational studies in Epidemiology
