# Correction: Effectiveness of a monthly schedule of follow-up for the treatment of uncomplicated severe acute malnutrition in Sokoto, Nigeria: A cluster randomized crossover trial

**DOI:** 10.1371/journal.pmed.1004370

**Published:** 2024-03-20

**Authors:** Matt D. T. Hitchings, Fatou Berthé, Philip Aruna, Ibrahim Shehu, Muhammed Ali Hamza, Siméon Nanama, Chizoba Steve-Edemba, Rebecca F. Grais, Sheila Isanaka

There are errors in the Funding and Competing Interests statements. The correct statements are:

**Funding:** Funding was provided to Epicentre, SI, and to MDTH by The Children’s Investment Fund Foundation. Médecins Sans Frontières (MSF) provided support for this study in the form of a salary for PA. The specific roles of this author are articulated in the ‘author contributions’ section. The funders had no role in study design, data collection and analysis, decision to publish, or preparation of the manuscript.

**Competing Interests:** The authors have read the journal’s policy and have the following competing interests to declare: Médecins Sans Frontières (MSF) provided support in the form of a salary for PA. RFG is an Academic Editor on *PLOS Medicine*’s editorial board. This does not alter our adherence to *PLOS Medicine* policies on sharing data and materials. There are no patents, products in development or marketed products associated with this research to declare.
