# Supplementary material for: Effectiveness of a monthly schedule of follow-up for the treatment of uncomplicated severe acute malnutrition in Sokoto, Nigeria: A cluster randomized crossover trial
Source: PLoS Med. 2022 Mar 1;19(3):e1003923. doi: 10.1371/journal.pmed.1003923 (PMC8887725; doi:10.1371/journal.pmed.1003923)
Supplement: S1 STROBE Checklist — STROBE, STrengthening the Reporting of OBservational studies in Epidemiology. (DOCX) [file pmed.1003923.s001.docx]

Effectiveness of a monthly schedule of follow-up for the treatment of uncomplicated severe acute malnutrition in Sokoto, Nigeria: a cluster-randomized crossover trial

**Supplementary Appendix 2**

Matt Hitchings, Fatou Berthé, Philip Aruna, Ibrahim Shehu, Muhammed Ali Hamza, Siméon Nanama, Chizoba Steve-Edemba, Rebecca F. Grais, and Sheila Isanaka

**Supplementary Appendix 2: CONSORT 2010 checklists**

**Table 1: CONSORT 2010 checklist of information to include when reporting a cluster randomised trial**

| Section/Topic | Item No | Standard Checklist item | Extension for cluster designs | Page No * |
| --- | --- | --- | --- | --- |
| Title and abstract | | | |  |
|  | 1a | Identification as a randomised trial in the title | Identification as a cluster randomised trial in the title | Title |
|  | 1b | Structured summary of trial design, methods, results, and conclusions (for specific guidance see CONSORT for abstracts)^[[1]](#endnote-1),^^[[2]](#endnote-2)^ | See table 2 | Abstract |
| Introduction | | | |  |
| Background and objectives | 2a | Scientific background and explanation of rationale | Rationale for using a cluster design | Introduction, Methods, paragraph 2 |
|  | 2b | Specific objectives or hypotheses | Whether objectives pertain to the cluster level, the individual participant level or both | Introduction |
| Methods | | | |  |
| Trial design | 3a | Description of trial design (such as parallel, factorial) including allocation ratio | Definition of cluster and description of how the design features apply to the clusters | Methods, paragraph 1, 2, 4 |
|  | 3b | Important changes to methods after trial commencement (such as eligibility criteria), with reasons |  | Methods, paragraph 6 |
| Participants | 4a | Eligibility criteria for participants | Eligibility criteria for clusters | Methods, paragraph 2 |
|  | 4b | Settings and locations where the data were collected |  | Methods, paragraph 2 |
| Interventions | 5 | The interventions for each group with sufficient details to allow replication, including how and when they were actually administered | Whether interventions pertain to the cluster level, the individual participant level or both | Methods, paragraph 5, 6, 8, 9, 10 |
| Outcomes | 6a | Completely defined pre-specified primary and secondary outcome measures, including how and when they were assessed | Whether outcome measures pertain to the cluster level, the individual participant level or both | Methods, paragraph 12-14 |
|  | 6b | Any changes to trial outcomes after the trial commenced, with reasons |  | N/A |
| Sample size | 7a | How sample size was determined | Method of calculation, number of clusters(s) (and whether equal or unequal cluster sizes are assumed), cluster size, a coefficient of intracluster correlation (ICC or *k*), and an indication of its uncertainty | Methods, paragraph 15 |
|  | 7b | When applicable, explanation of any interim analyses and stopping guidelines |  | N/A |
| Randomisation: | | | |  |
| Sequence generation | 8a | Method used to generate the random allocation sequence |  | Methods, paragraph 11 |
|  | 8b | Type of randomisation; details of any restriction (such as blocking and block size) | Details of stratification or matching if used | Methods, paragraph 11 |
| Allocation concealment mechanism | 9 | Mechanism used to implement the random allocation sequence (such as sequentially numbered containers), describing any steps taken to conceal the sequence until interventions were assigned | Specification that allocation was based on clusters rather than individuals and whether allocation concealment (if any) was at the cluster level, the individual participant level or both | Methods, paragraph 11 |
| Implementation | 10 | Who generated the random allocation sequence, who enrolled participants, and who assigned participants to interventions | Replace by 10a, 10b and 10c | Methods, paragraph 11 |
|  | 10a |  | Who generated the random allocation sequence, who enrolled clusters, and who assigned clusters to interventions | Methods, paragraph 11 |
|  | 10b |  | Mechanism by which individual participants were included in clusters for the purposes of the trial (such as complete enumeration, random sampling) | Methods, paragraph 11 |
|  | 10c |  | From whom consent was sought (representatives of the cluster, or individual cluster members, or both), and whether consent was sought before or after randomisation | Methods, paragraph 11 |
|  |  |  |  |  |
| Blinding | 11a | If done, who was blinded after assignment to interventions (for example, participants, care providers, those assessing outcomes) and how |  | N/A |
|  | 11b | If relevant, description of the similarity of interventions |  | N/A |
| Statistical methods | 12a | Statistical methods used to compare groups for primary and secondary outcomes | How clustering was taken into account | Methods, paragraph 16 |
|  | 12b | Methods for additional analyses, such as subgroup analyses and adjusted analyses |  | Methods, paragraph 16 |
| Results | | | |  |
| Participant flow (a diagram is strongly recommended) | 13a | For each group, the numbers of participants who were randomly assigned, received intended treatment, and were analysed for the primary outcome | For each group, the numbers of clusters that were randomly assigned, received intended treatment, and were analysed for the primary outcome | Results, paragraph 1 and Fig 1 |
|  | 13b | For each group, losses and exclusions after randomisation, together with reasons | For each group, losses and exclusions for both clusters and individual cluster members | Results, paragraph 1 and Fig 1 |
| Recruitment | 14a | Dates defining the periods of recruitment and follow-up |  | Results, paragraph 1 |
|  | 14b | Why the trial ended or was stopped |  | Results, paragraph 1 and Fig 1 |
| Baseline data | 15 | A table showing baseline demographic and clinical characteristics for each group | Baseline characteristics for the individual and cluster levels as applicable for each group | Table 1 |
| Numbers analysed | 16 | For each group, number of participants (denominator) included in each analysis and whether the analysis was by original assigned groups | For each group, number of clusters included in each analysis | Results, paragraph 1 |
| Outcomes and estimation | 17a | For each primary and secondary outcome, results for each group, and the estimated effect size and its precision (such as 95% confidence interval) | Results at the individual or cluster level as applicable and a coefficient of intracluster correlation (ICC or k) for each primary outcome | Results, paragraph 3-9 |
|  | 17b | For binary outcomes, presentation of both absolute and relative effect sizes is recommended |  | N/A |
| Ancillary analyses | 18 | Results of any other analyses performed, including subgroup analyses and adjusted analyses, distinguishing pre-specified from exploratory |  | Results, paragraph 3-9 |
| Harms | 19 | All important harms or unintended effects in each group (for specific guidance see CONSORT for harms^[[3]](#endnote-3)^) |  | Results, paragraph 3-9 |
| Discussion | | | |  |
| Limitations | 20 | Trial limitations, addressing sources of potential bias, imprecision, and, if relevant, multiplicity of analyses |  | Discussion, paragraph 7 |
| Generalisability | 21 | Generalisability (external validity, applicability) of the trial findings | Generalisability to clusters and/or individual participants (as relevant) | Discussion, paragraph 7 |
| Interpretation | 22 | Interpretation consistent with results, balancing benefits and harms, and considering other relevant evidence |  | Discussion, paragraph 9 |
| Other information | | |  |  |
| Registration | 23 | Registration number and name of trial registry |  | Methods, paragraph 1 |
| Protocol | 24 | Where the full trial protocol can be accessed, if available |  | Supplement |
| Funding | 25 | Sources of funding and other support (such as supply of drugs), role of funders |  | With application |

** Note: page numbers optional depending on journal requirements*

| **Section/topic** | **Item No** | **Description** | **Page No*** |
| --- | --- | --- | --- |
| Title† | 1a | Identification as a randomised crossover trial in the title | Title |
| Abstract† | 1b | Specify a crossover design and report all information outlined in table 2 | Abstract |
| Introduction: | | | |
| Background‡ | 2a | Scientific background and explanation of rationale | Introduction, Methods, paragraph 2 |
| Objectives‡ | 2b | Specific objectives or hypotheses | Introduction |
| Methods: | | | |
| Trial design† | 3a | Rationale for a crossover design. Description of the design features including allocation ratio, especially the number and duration of periods, duration of washout period, and consideration of carry over effect | Methods, paragraph 1, 2, 4 |
| Change from protocol‡ | 3b | Important changes to methods after trial commencement (such as eligibility criteria), with reasons | Methods, paragraph 6 |
| Participants‡ | 4a | Eligibility criteria for participants | Methods, paragraph 2 |
| Settings and location‡ | 4b | Settings and locations where the data were collected | Methods, paragraph 2 |
| Interventions† | 5 | The interventions with sufficient details to allow replication, including how and when they were actually administered | Methods, paragraph 5, 6, 8, 9, 10 |
| Outcomes‡ | 6a | Completely defined prespecified primary and secondary outcome measures, including how and when they were assessed | Methods, paragraph 12-14 |
| Changes to outcomes‡ | 6b | Any changes to trial outcomes after the trial commenced, with reasons | N/A |
| Sample size† | 7a | How sample size was determined, accounting for within participant variability | Methods, paragraph 15 |
| Interim analyses and stopping guidelines‡ | 7b | When applicable, explanation of any interim analyses and stopping guidelines | N/A |
| Randomisation: | | | |
| Sequence generation‡ | 8a | Method used to generate the random allocation sequence | Methods, paragraph 11 |
| Sequence generation‡ | 8b | Type of randomisation; details of any restriction (such as blocking and block size) | Methods, paragraph 11 |
| Allocation concealment mechanism‡ | 9 | Mechanism used to implement the random allocation sequence§ (such as sequentially numbered containers), describing any steps taken to conceal the sequence until interventions were assigned | Methods, paragraph 11 |
| Implementation† | 10 | Who generated the random allocation sequence,§ who enrolled participants, and who assigned participants to the sequence of interventions | Methods, paragraph 11 |
| Blinding‡ | 11a | If done, who was blinded after assignment to interventions (for example, participants, care providers, those assessing outcomes) and how | N/A |
| Similarity of interventions‡ | 11b | If relevant, description of the similarity of interventions | N/A |
| Statistical methods† | 12a | Statistical methods used to compare groups for primary and secondary outcomes which are appropriate for crossover design (that is, based on within participant comparison) | Methods, paragraph 16 |
| Additional analyses‡ | 12b | Methods for additional analyses, such as subgroup analyses and adjusted analyses | Methods, paragraph 16 |
| Results | | | |
| Participant flow (a diagram is strongly recommended)† | 13a | The numbers of participants who were randomly assigned, received intended treatment, and were analysed for the primary outcome, separately for each sequence and period | Results, paragraph 1 and Fig 1 |
| Losses and exclusions† | 13b | No of participants excluded at each stage, with reasons, separately for each sequence and period | Results, paragraph 1 and Fig 1 |
| Recruitment‡ | 14a | Dates defining the periods of recruitment and follow-up | Results, paragraph 1 |
| Trial end‡ | 14b | Why the trial ended or was stopped | Results, paragraph 1 and Fig 1 |
| Baseline data† | 15 | A table showing baseline demographic and clinical characteristics by sequence and period | Table 1 |
| Numbers analysed† | 16 | Number of participants (denominator) included in each analysis and whether the analysis was by original assigned groups | Results, paragraph 1 |
| Outcomes and estimation† | 17a | For each primary and secondary outcome, results including estimated effect size and its precision (such as 95% confidence interval) should be based on within participant comparisons.¶ In addition, results for each intervention in each period are recommended | Results, paragraph 3-9 |
| Binary outcomes‡ | 17b | For binary outcomes, presentation of both absolute and relative effect sizes is recommended | N/A |
| Ancillary analyses‡ | 18 | Results of any other analyses performed, including subgroup analyses and adjusted analyses, distinguishing prespecified from exploratory | Results, paragraph 3-9 |
| Harms† | 19 | Describe all important harms or untended effects in a way that accounts for the design (for specific guidance, see CONSORT for harms32) | Results, paragraph 3-9 |
| Discussion: | | | |
| Limitations† | 20 | Trial limitations, addressing sources of potential bias, imprecision, and if relevant, multiplicity of analyses. Consider potential carry over effects | Discussion, paragraph 7 |
| Generalisability‡ | 21 | Generalisability (external validity, applicability) of the trial findings | Discussion, paragraph 7 |
| Interpretation‡ | 22 | Interpretation consistent with results, balancing benefits and harms, and considering other relevant evidence | Discussion, paragraph 9 |
| Other information: | | | |
| Registration‡ | 23 | Registration number and name of trial registry | Methods, paragraph 1 |
| Protocol‡ | 24 | Where the full trial protocol can be accessed, if available | Supplement |
| Funding‡ | 25 | Sources of funding and other support (such as supply of drugs), role of funders | With application |

1. Hopewell S, Clarke M, Moher D, Wager E, Middleton P, Altman DG, et al. CONSORT for reporting randomised trials in journal and conference abstracts. *Lancet* 2008, 371:281-283 [↑](#endnote-ref-1)
2. Hopewell S, Clarke M, Moher D, Wager E, Middleton P, Altman DG at al (2008) CONSORT for reporting randomized controlled trials in journal and conference abstracts: explanation and elaboration. *PLoS Med* 5(1): e20 [↑](#endnote-ref-2)
3. Ioannidis JP, Evans SJ, Gotzsche PC, O'Neill RT, Altman DG, Schulz K, Moher D. Better reporting of harms in randomized trials: an extension of the CONSORT statement. *Ann Intern Med* 2004; 141(10):781-788. [↑](#endnote-ref-3)
