# Supplementary material for: Effectiveness of a monthly schedule of follow-up for the treatment of uncomplicated severe acute malnutrition in Sokoto, Nigeria: A cluster randomized crossover trial
Source: PLoS Med. 2022 Mar 1;19(3):e1003923. doi: 10.1371/journal.pmed.1003923 (PMC8887725; doi:10.1371/journal.pmed.1003923)
Supplement: S1 Protocol — (DOCX) [file pmed.1003923.s002.docx]

Reducing the frequency of follow up and task sharing in the treatment of uncomplicated severe acute malnutrition: an evaluation of monthly visits and home-based surveillance for access-limited and high-burden settings

December 16, 2017

Version 1.5

| Title | Reducing the frequency of follow up and task sharing in the treatment of uncomplicated severe acute malnutrition: an evaluation of monthly visits and home-based surveillance for access-limited and high-burden settings |
| --- | --- |
| Study Site | Sokoto, Nigeria |
| Principal Investigator and Corresponding Investigator | Sheila Isanaka, ScD  Harvard T.H. Chan School of Public Health  655 Huntington Avenue  Boston, MA 02115  Tel : + 1 617 432 4623  Email : [sisanaka@hsph.harvard.edu](mailto:sisanaka@hsph.harvard.edu) |
| Co-investigators | Rebecca Grais, PhD  Epicentre  Tel : +33 1 4021 5475  Email: rebecca.grais@epicentre.msf.org |
| Field Investigator | Fatou Berthé, MD  Epicentre Niger  Tel : +277 9219 9442  Email: fatou.berthe@epicentre.msf.org |
| Operational Partners | Sokoto State Nutrition Team  Epicentre  UNICEF Nigeria |
| Protocol Version | Version number 1.5 (Issue date: December 16 , 2017) |

Abstract (plain language summary)

**Title:** Reducing the frequency of follow up and task sharing in the treatment of uncomplicated severe acute malnutrition: an evaluation of monthly visits and home-based surveillance for access-limited and high-burden settings

Research design: This study will be conducted as a stratified cluster randomized trial. The unit of randomization will be the outpatient therapeutic feeding center. The 10 health centers will be stratified by size, and centers within a stratum will be randomized in a 1:1 ratio to one of two schedules of treatment: (1) standard weekly visits or (2) monthly visits with support for home-based surveillance.

Methodology: Distribution of the therapeutic feeding rations, as well as medical and anthropometric surveillance of children being treated for uncomplicated severe acute malnutrition, will take place on a weekly or monthly basis until discharge according to the random assignment of the site. Caregivers in the monthly visit group will receive additional instruction at admission regarding home-based surveillance.

Routine program data on the child’s anthropometric and clinical status at scheduled follow-up visits will be collected on standardized case report forms. All children will receive additional visits within the first two weeks of treatment and 3months post-discharge to assess the child’s anthropometric status.

Principal intervention:

1. standard weekly follow-up during treatment for uncomplicated severe acute malnutrition
2. monthly follow-up with support for home-based surveillance

Regardless of intervention assignment, all children with uncomplicated severe acute malnutrition will receive standard medical care as per national guidelines.

Outcome variables:

- Nutritional recovery, defined by being free from medical complications, MUAC ≥ 125 mm, and no edema for 2 weeks if admitted with edema.
- Hospitalization, defined as referral to inpatient care for weight/edema changes; failed appetite test; or clinical complication necessitating inpatient care.
- Weight gain (g / kg / day), defined as the difference in weight (g) from admission to week 4, to week 8 and to discharge divided by weight on admission (kg) and number of days between admission and week 4/week8/discharge among recovered children.
- Defaulting, defined as 3 missed scheduled facility visits in the weekly follow-up group and 1 missed scheduled facility visit in the monthly follow-up group.
- Relapse, defined as maternal report of admission to any therapeutic feeding program within 3 months of discharge from the index admission.

TABLE OF CONTENTS

[1.0 BACKGROUND AND RATIONALE 6](#_Toc501054239)

[1.1 Management of severe acute malnutrition 6](#_Toc501054240)

[1.2 Study rationale 7](#_Toc501054241)

[1.3 Innovation and relevance for the community-based management of SAM 7](#_Toc501054242)

[1.4 Preliminary studies 8](#_Toc501054243)

[2.0 STUDY OBJECTIVES AND ENDPOINTS 9](#_Toc501054244)

[3.0 METHODOLOGY 11](#_Toc501054245)

[3.1 Study design 11](#_Toc501054246)

[3.2 Study setting 11](#_Toc501054247)

[3.3. Study sites 11](#_Toc501054248)

[3.4 Target population 12](#_Toc501054249)

[3.5 Study intervention 13](#_Toc501054250)

[3.6 Random allocation and concealment 13](#_Toc501054251)

[3.7 Power calculations 13](#_Toc501054252)

[3.8 Data entry, management and quality control 14](#_Toc501054253)

[3.9 Statistical analysis 14](#_Toc501054254)

[3.10 Cost analysis 15](#_Toc501054255)

[4.0 STUDY PROCEDURES AND SCHEDULE 16](#_Toc501054256)

[4.1 Community presentation and engagement 16](#_Toc501054257)

[4.2 Recruitment schedule 16](#_Toc501054258)

[4.3 Evaluation of eligibility 16](#_Toc501054259)

[4.4 Informed consent 17](#_Toc501054260)

[4.5 Completion of admission visit 17](#_Toc501054261)

[4.6 Follow-up visits 18](#_Toc501054262)

[4.7 Unannounced safety visits within 2 weeks following inclusion 19](#_Toc501054263)

[4.8 Tracing for missed visits (absences) and defaulting 19](#_Toc501054264)

[4.9 Post-discharge follow-up 20](#_Toc501054265)

[4.10 Standard care 20](#_Toc501054266)

[4.11 Timeline 21](#_Toc501054267)

[5.0 ETHICAL CONSIDERATIONS 22](#_Toc501054268)

[5.1 Summary of known and potential risks 22](#_Toc501054269)

[5.2 Risk minimization and benefits 22](#_Toc501054270)

[5.3 Informed consent 23](#_Toc501054271)

[5.4 Confidentiality 24](#_Toc501054272)

[5.5 Reimbursement 24](#_Toc501054273)

[5.6 Research-related injuries 24](#_Toc501054274)

[5.7 Storage of specimens 25](#_Toc501054275)

[5.8 Institutional Review Board approval 25](#_Toc501054276)

[5.9 Reportable events 25](#_Toc501054277)

[5.10 Declaration of conflict of interests 25](#_Toc501054278)

[5.11 Trial registration 25](#_Toc501054279)

[5.12 Regulatory compliance 25](#_Toc501054280)

[6.0 PARTNERSHIPS 26](#_Toc501054281)

[7.0 ADMINISTRATIVE AND LOGISTICAL CONSIDERATIONS 27](#_Toc501054282)

[7.1 Human resources 27](#_Toc501054283)

[7.2 Logistics 27](#_Toc501054284)

[8.0 REFERENCES 28](#_Toc501054285)

[9.0 ANNEXES 29](#_Toc501054286)

# 1.0 BACKGROUND AND RATIONALE

## 1.1 Management of severe acute malnutrition

Severe acute malnutrition (SAM) affects at least 19 million children worldwide, and through its detrimental effects on immune function, contributes to high child morbidity and mortality.^1^ Historically, the treatment of children with SAM included the systematic hospitalization of all cases and inpatient nutritional rehabilitation with milk-based diets over 3-4 weeks.^2^ In 2007, a joint United Nations statement endorsed a new model for the management of SAM that combines outpatient treatment with ready-to-use therapeutic foods (RUTF) for uncomplicated cases and inpatient treatment only for complicated cases.^3^ In this model, 80 to 90% of children with uncomplicated SAM are treated on a weekly outpatient basis, where they receive rapid health assessment and weight check by a nurse, a physical exam if signs of illness or weight loss are present, and a therapeutic ration of RUTF for home use. The shift from systematic inpatient to outpatient-based care has decreased the need for highly trained medical staff and inpatient bed capacity, reduced the risk of cross-infection among ill children, and importantly eliminated the burden on caregivers and children spending several weeks in hospital.^4^ Although outpatient care places increased responsibility on caregivers to supervise administration of RUTF at home and monitor the child’s clinical condition between weekly visits, low mortality rates (e.g. 1 to 5 %) have been achieved.

While the outpatient treatment model represents great progress in the management of SAM, the current model remains impractical in some contexts, particularly in settings where geography (e.g. the south of Chad), security (e.g. Somalia) or nomadic livelihoods (e.g. Kenya) make frequent movements to a health facility difficult or impossible. In settings with a high incidence of acute malnutrition (e.g. Niger and Northeastern Nigeria), such frequent visits may also overwhelm public health capacity, resulting in reduced coverage of treatment. Therefore, in many settings, it may be necessary to identify an alternative delivery model for the outpatient management of uncomplicated SAM.

While there is some operational experience with biweekly schedules of follow up, evidence for further reduced schedules in the outpatient treatment of SAM is extremely limited. To our knowledge, the only experience to date comes from the 2012 Medécins Sans Frontières (MSF)-France project in Yao, Chad, where an active screening and treatment program using mobile teams extended the standard, weekly schedule of follow up to a monthly schedule (personal communication, S. Shepherd). Unfortunately, flooding in the program area reduced access and forced the closing of treatment sites, limiting our understanding of the feasibility and effectiveness of this model.

If a reduced schedule of follow up, with caregiver support for home-based surveillance, is to be adopted where standard weekly follow up is not possible, there must be clear evidence that such strategies can be used safely and effectively while remaining acceptable to caregivers. A reduced, monthly schedule of follow up has the potential to reduce the opportunity cost of caregivers seeking treatment, increase access to adequate care and support among vulnerable children, and allow for more efficient delivery in numerous contexts.

## 1.2 Study rationale

While it is estimated that at least 19 million children suffer from SAM each year, only 7-13% of these children received treatment in 2012.^5^ The challenges to increasing access to treatment are multiple, but reliance on a weekly schedule of follow up can limit operations to areas where access and frequent visits to a health facility are not possible. In contexts where weekly follow up is not feasible, humanitarian organizations may be forced to curtail or limit operations, resulting in fewer children receiving life-saving treatment and care and reducing the impact of nutritional programs in these settings.

To our knowledge, there has been no high quality research on the use of alternative schedules in the treatment of uncomplicated SAM among young children. Important research gaps in the evidence base therefore remain, including a lack of information on the feasibility, acceptability, safety and effectiveness of alternative delivery models. This study will apply the gold standard design for evaluation, with the aim to produce evidence of sufficient quality and rigor to fill the existing research gap and inform future programs. These results will be drawn from an on-going nutritional program, and therefore aim to be directly generalizable to other operational settings where the standard protocol may be impractical. This includes contexts with ongoing conflict or security constraints, as well as settings with difficult geographical access and a high incidence of acute malnutrition. The operational experience provided by this research will also be an opportunity to better understand the type of support (e.g. home visits to follow absences, increased support/training of caregivers) that would be necessary for a monthly schedule of follow up to be feasible and eventually applicable in other contexts.

## 1.3 Innovation and relevance for the community-based management of SAM

The outpatient management of SAM has been recommended by the World Health Organization, WFP, UN Standing Committee on Nutrition and UNICEF since 2007. However, reliance on a weekly schedule of follow up limits operations to areas where access and frequent visits to a health facility are possible and potentially reduces the number of children receiving treatment. In contexts where weekly follow up is not feasible (e.g. Chad where access was limited due to heavy rains; Somalia where access is reduced due to high insecurity), programs have been forced to curtail operations, resulting in fewer children receiving treatment. Similarly in contexts where the absolute number of children needing treatment is very high (e.g. northeastern Nigeria), the burden of the traditional schedule of follow up can limit coverage and reduce program impact. To our knowledge, no program has implemented a reduced schedule of follow up (beyond biweekly visits) and documented safety or efficacy. This proposal therefore represents innovation as this is the first time a strategic reduction in the schedule of follow up will be implemented and evaluated within the community-based treatment model.

This study is intended to provide programs with greater flexibility to consider more innovative models of treatment and to extend operations into areas previously unsuitable for treatment programs. If the results of this study can confirm that monthly visits in the treatment of uncomplicated SAM can achieve similar rates of nutritional recovery as standard weekly visits, programs will have the evidence needed to consider adopting a reduced schedule of follow up and provide treatment in a greater number of settings. Evidence provided by this study could support increased coverage in existing programs, as a reduced schedule of follow up in an existing program could decrease the program burden per child and allow for an increase in the proportion of children covered using the same or fewer resources. These results could also allow more flexible and innovative programming in new settings where the standard protocol has been impractical, including contexts with nomadic populations, difficult geographical access, conflict or high incidence of malnutrition.

## 1.4 Preliminary studies

The proposed monthly schedule of follow up will involve an extension of the period between which the child receives a health assessment by a health worker. During this time, caregivers will be asked to monitor the clinical and anthropometric status of the child at home and return to the health facility for medical attention upon development of any clinical sign of concern.

To support caregivers in these tasks, culturally-appropriate educational tools and messages, including materials for facility-based instruction (e.g. posters, pictorial flip charts), as well as for home-based use (e.g. pocket pictorial cards), were developed with MSF-France and the LASDEL in 2014. Tools covered the key topics of: a) home-based MUAC measurement; b) clinical surveillance of key danger signs or symptoms; and c) appropriate storage and distribution of the monthly therapeutic ration.

A mixed-methods pilot study of the educational tools and key messages, as well as feasibility of a monthly RUTF ration, was completed in 2015 in Madarounfa, Niger. The validity and reliability of caregiver MUAC measurements, and the retention of key messages, were assessed using standardized questionnaires administered pre- and post- intervention. Weight gain and unannounced home spot checks were used to assess home utilization of the monthly ration. Focus group discussions and semi-structured interviews with caregivers, heads of households, and health workers were used to assess the suitability of the educational tools and key messages. Results from this pilot study suggested that 1) caregivers satisfactorily retained information on clinical danger signs and the correct method to measure MUAC immediately and 28-days following a brief training and 2) the provision of a monthly ration of RUTF can be both feasible and safe, with the correct home storage and utilization of RUTF observed in the majority of households visited early in treatment, and very few cases of weight loss or hospitalization (results in press).

# 2.0 STUDY OBJECTIVES AND ENDPOINTS

The study aims to strengthen the evidence base for more flexible ways to deliver nutritional treatment in humanitarian crises.

Primary objective:

To estimate the effectiveness of monthly vs. standard weekly follow up in the treatment of uncomplicated severe acute malnutrition in children aged 6 to 59 months in terms of nutritional recovery.

Secondary objectives:

- To estimate the effectiveness of monthly vs. standard weekly follow up in the treatment of uncomplicated severe acute malnutrition in children aged 6 to 59 months in terms of hospitalization.
- To estimate the effectiveness of monthly vs. standard weekly follow up in the treatment of uncomplicated severe acute malnutrition in children aged 6 to 59 months in terms of weight gain among recovered children.
- To estimate the effect of monthly vs. standard weekly follow up in the treatment of uncomplicated severe acute malnutrition in children aged 6 to 59 months in terms of the risk of defaulting.
- To estimate the effectiveness of monthly vs. standard weekly follow up in the treatment of uncomplicated severe acute malnutrition in children aged 6 to 59 months in terms of the risk of relapse within 3 months following discharge.
- To estimate program coverage in areas receiving monthly vs. standard weekly follow up in the treatment of uncomplicated severe acute malnutrition in children aged 6 to 59.
- To assess the cost-effectiveness of monthly vs. standard weekly follow up in the treatment of uncomplicated severe acute malnutrition in children aged 6 to 59 months in terms of cost per child treated and per child recovered.

Primary endpoint:

The primary endpoint is nutritional recovery, defined by being free from medical complications, MUAC ≥ 125 mm, and no edema for 2 weeks if admitted with edema.

Secondary endpoints:

- Hospitalization, defined as referral to inpatient care for :
  - weight/edema changes: weight loss over 3 weeks; static weight over 4 weeks; onset of edema when previously absent; persistent edema 3 weeks following admission
  - failed appetite test
  - clinical complication necessitating inpatient care: fever, hypothermia, severe dehydration, repeated vomiting, severe respiratory distress, chest in-drawing, severe pallor with respiratory distress, malaria with signs of severity, abscess or extended skin lesions, very weak, apathy or unconscious, or convulsions
- Weight gain (g / kg / day), defined as the difference in weight (g) from admission to week 4, to week 8 and to discharge divided by weight on admission (kg) and number of days between admission and week 4/week 8/discharge among recovered children.
- Defaulting, defined as 3 missed scheduled facility visits in the weekly follow-up group and 1 missed scheduled facility visit in the monthly follow-up group.
- Relapse, defined as maternal report of admission to any therapeutic feeding program within 3 months of discharge from the index admission.
- Program coverage, defined as single coverage of SAM treatment, estimated in the weekly and monthly follow-up groups using the SQUEAC methodology.
- Cost per child treated and per child recovered, defined using the societal perspective to identify all provider and caregiver costs associated with treatment.

# 3.0 METHODOLOGY

## 3.1 Study design

This study will be conducted as a stratified cluster randomized trial of a monthly schedule of follow up in the treatment of uncomplicated SAM among children aged 6 to 59 months. The unit of randomization will be the outpatient therapeutic feeding center. The 10 health centers supported by UNICEF will be stratified by size (± 500 admissions per site), and centers within a stratum will be randomized in a 1:1 ratio to one of two schedules of treatment: (1) standard weekly visits or (2) monthly visits with support for home-based surveillance.

Distribution of the therapeutic feeding rations, as well as medical and anthropometric surveillance, will take place on a weekly or monthly basis until discharge according to the random assignment of the site. Caregivers in the monthly visit group will receive additional instruction at admission regarding home-based MUAC measurement and clinical surveillance.

Regardless of intervention assignment, all children with uncomplicated SAM will receive standard medical care on admission as per national guidelines, and a home visit 3 months following discharge from the nutritional program. Additional data collection, including coverage assessment and economic costing analysis will be used to address the secondary objectives related to the evaluation of coverage and cost-effectiveness, respectively.

## 3.2 Study setting

The study is proposed to take place in the Binji and Wamako local government areas (LGA) within Sokoto state of Northwestern Nigeria. This location represents a reasonable location for this study within an on-going nutritional program. As the study will be conducted over an extended period of time, the relative stability of this location is also expected to provide secure, uninterrupted access to the study population for monitoring and data collection. Finally, this setting includes a rural area largely representative of the Sahel region with endemic acute malnutrition and medium population density, where a reduced schedule of follow up would be justified and provide an operational advantage. It also represents settings of chronic food security crisis, where the rates of malnutrition are important. In 2015, the National Nutrition and Health Survey reported a prevalence of Global Acute Malnutrition (GAM) and SAM of 14.2% and 2.7%, respectively, when measured by MUAC among children < 5 years of age in Sokoto.^6^ Evidence drawn from this chronic crisis can therefore be expected to provide evidence relevant to other settings of the Sahel and beyond.

The study will benefit from partnerships with Epicentre and UNICEF Nigeria – both of whom have extensive an established presence and experience in the study region, and will advise the study on local culture and customs.

## 3.3. Study sites

Study activities will take place in 10 outpatient therapeutic feeding centers supported by UNICEF. The 10 outpatient therapeutic feeding centers will be staffed by research nurses trained to seek written informed consent and support data collection during all enrollment and follow-up visits. Children transferred for inpatient care will be followed by a research nurse until hospital discharge. Once medical complications are resolved, appetite has returned and/or edema is reduced, the child will resume treatment at the original outpatient therapeutic feeding center and study follow-up until program discharge.

## 3.4 Target population

The study subjects will be children newly admitted for the treatment of uncomplicated SAM at one of 10 outpatient therapeutic feeding centers.

The specific eligibility criteria for the treatment of uncomplicated SAM:

- age from 6 to 59 months
- MUAC < 115 mm and/or grade 1-2 edema
- Absence of current illness requiring inpatient care*

*As per current treatment protocols, children will be referred to inpatient care if presenting with lack of appetite, intractable/continuous vomiting, fever, hypothermia, lower respiratory tract infection, severe respiratory distress, any chest in-drawing, severe anemia (hemoglobin < 7 g/dl), skin infection or lesions requiring IM/IV treatment, altered consciousness, hypoglycaemia, convulsions, severe dehydration, presence of grade 3 edema.

The specific inclusion criteria for the study are:

- Eligible for new admission** at one of the study outpatient therapeutic feeding centers
- Written informed consent of parent or legal guardian

**New admissions include cases of relapse, where the child was previously successfully treated, discharged as cured and returns with a new episode of acute malnutrition within 2 months of discharge. New admission will include children transferred from inpatient care to outpatient care to continue treatment but does not include children transferred from another outpatient site. New admission does not include returned defaulters who were absent for 3 consecutive visits before recovery and return to continue treatment.

The specific exclusion criteria for the study are:

- History of allergy to peanuts
- Any other condition in which, in the judgment of the Field Investigator, would interfere with or serves as a contraindication to protocol adherence or the ability to give informed consent***

***While determining eligibility, study staff will be asked to use good clinical judgment in considering a participant’s overall suitability for inclusion. Some participants may not be appropriate for the study even if they meet all inclusion criteria. For instance, medical, occupational or other conditions of the participant or parent/guardian may make routine home visits and evaluation difficult or make the child a poor candidate for retention. Any such potential exclusion must be validated by the Field Investigator.

## 3.5 Study intervention

The study will compare two schedules of follow-up:

1. Standard weekly visits at the outpatient therapeutic feeding center until discharge
2. Monthly visits at the outpatient therapeutic feeding center with caregiver support for home-based surveillance, with visits scheduled at weeks 4, 8, 10 and 12 until discharge.

Regardless of intervention assignment, on admission, all children will receive standard medical care as per national guidelines. On admission, caregivers with children admitted to a center randomized to monthly visits will also receive additional instruction and take home materials regarding home-based MUAC and clinical surveillance. All educational materials were developed in consultation with MSF-France, adapted in the field with the LASDEL and pilot tested by Epicentre in Niger in 2014. Draft study materials (provided in Annex 9.1) will be tested and adapted to the study context prior to the start of any study activities in Nigeria.

At all scheduled follow-up visits (either weekly or monthly), children will receive a physical examination and anthropometric assessment. All caregivers will be instructed to return to the health center between scheduled follow up visits if any clinical sign of concern develops.

## 3.6 Random allocation and concealment

The reduced schedule of follow-up will be tested using a stratified cluster randomized design, with the health center as the unit of randomization. All children admitted for outpatient therapeutic feeding at a participating health center will receive the same schedule of follow-up.

Health centers will be stratified by size according to the number of admissions per site in order to promote balance of the intervention among children. Centers within a stratum will be randomized in 1:1 ratio to standard weekly vs. monthly follow-up. Randomization assignment will be made by lottery, in which a local representative will select the name of one of the two interventions from an envelope or opaque jar.

The study will be open, as it will not be possible to blind participants or study staff to frequency of follow up received.

## 3.7 Power calculations

Ten outpatient therapeutic feeding centers in this setting can be expected to admit total of 3,500 children for the treatment of uncomplicated SAM over 12 months (3,777 children admitted at 10 facilities in 2016). Assuming a baseline risk of nutritional recovery in the weekly follow-up group of 0.80, intracluster correlation of 0.002 and significance level of 0.05 using the one-sided Score test,^7^ and allowing for 20% loss to follow up, a sample size of 350 children per health facility (2100 per group) will achieve 88% power to detect a non-inferiority margin difference between the group proportions of -0.05.

|  | Difference in recovery between weekly vs. monthly follow up groups (n = 350 per site) | | |
| --- | --- | --- | --- |
| Intracluster correlation | Δ = -0.15 | Δ = -0.10 | Δ = -0.05 |
| 0.001 | 1.00 | 1.00 | 0.94 |
| 0.002 | 1.00 | 1.00 | 0.88 |
| 0.01 | 1.00 | 0.97 | 0.54 |

## 3.8 Data entry, management and quality control

All of the information required by the study protocol will be entered on standardized case report forms (CRF) provided by the principal investigator (provided in Annex 9.2). The study nurses assigned to each health center and Field Investigator will validate all CRFs for completeness and accuracy, signing and dating each to attest to his/her responsibility for the quality of all data recorded and that the data represents a complete and accurate record of each child’s participation in the study. All validated CRFs will be double-entered in an electronic database, compared and verified for accuracy. Data quality will be enforced through a variety of mechanisms, including referential data rules, valid values, range checks, and consistency checks against data already stored in the database (i.e., longitudinal checks).

A data validation plan will be prepared by the Principal Investigator. Errors will be detected by programs designed to detect missing data or specific errors in the data. These errors will be summarized along with detailed descriptions of the specific problem in a Data Query Report, which will be sent to the Field Investigator for resolution by checking the original forms for inconsistency, checking other sources to determine the correction, and modifying the original (paper) form as appropriate. Written documentation of changes will be available via electronic logs. A complete back up of the study database will be performed twice a month; incremental data back-ups will be performed on a daily basis. The data manager will provide monthly email reports with information on missing data, missing forms, and missing visits to the sponsor. Epicentre alone will have full access to the data.

## 3.9 Statistical analysis

The primary outcome is nutritional recovery. Secondary outcomes include hospitalization, average daily weight gain among recovered children, defaulting, and relapse within 3 months.

All analyses will use the intention-to-treat principle and account for the stratified cluster design. We will use generalized estimating equations and robust standard errors to compare the proportion of children recovered, hospitalized, defaulted or relapsed within 3 months, and mixed effects models to compare average daily weight gain.

Interim analysis at 6 months of inclusion will be conducted to ensure the safety of children included. No adjustment will be made for multiple testing. All P values will be 2-sided, with P < 0.05 considered statistically significant. Analyses will be performed using SAS version 9.3 or higher (SAS Institute Inc., Cary, NC).

## 3.10 Cost analysis

Using the broadest ‘societal’ perspective, we will estimate the total cost of treatment per child treated and per child recovered. The relevant types of costs will include direct medical costs [provider time, capital costs (building space, equipment), consumables (medical supplies, medications), and overhead (electricity, water, and maintenance)], direct non-medical costs [fees for participant travel, food, lodging, and child care], and indirect costs [time spent seeking care] during both inpatient and outpatient care. Data on resources used will be collected on standardized forms and supplemented with key informant interviews with study staff and accounting records. An incremental cost-effectiveness ratio (ICER) will be calculated as the difference in cost between groups divided by the difference in effectiveness between treatment strategies, expressed in dollars per unit of effect (e.g. per case treated, case recovered).

# 4.0 STUDY PROCEDURES AND SCHEDULE

## 4.1 Community presentation and engagement

To facilitate broad understanding and acceptance of the study before implementation, study staff will meet with key individuals in all study villages, heads of all households, religious leaders, community health workers and elderly women, to provide detailed information on the study objectives and procedures prior to the start of enrollment. Presentation of the study in this manner is intended to support high levels of participation and retention in study activities.

## 4.2 Recruitment schedule

Recruitment will take place in 10 outpatient therapeutic feeding centers over a minimum of 12 months.

## 4.3 Evaluation of eligibility

Evaluation of eligibility will occur at the time of first visit/admission to the study outpatient therapeutic feeding centers. Upon arrival, program staff will screen children for eligibility for outpatient treatment as per standard protocol (e.g. age 6 to 59 months; MUAC < 115 mm or grade 1-2 edema; and absence of illness requiring inpatient management). Anthropometry (weight to the nearest 100 g; length (children <24 months) or stature (children ≥ 24 months) to the nearest 0.1 cm; and MUAC to the nearest 0.1 cm using the UNICEF band) will be assessed by program staff using standard techniques described for the WHO Multicenter Growth Reference Study. All study and program staff will be trained and standardized prior to beginning data collection. Standardization sessions will be repeated every 3 months during data collection. Children ineligible for outpatient treatment will not be admitted for nutritional therapy and asked to return home, with explanation of the program admission criteria. These children may be referred to the primary pediatric care program or vaccination program, as needed.

If the child is eligible for outpatient treatment, a study nurse will evaluate the specific study inclusion and exclusion criterion (e.g. new admission, no peanut allergy). If a child presenting to a site assigned to weekly follow-up is eligible for SAM treatment but found ineligible for study inclusion, the child will receive all medical and nutritional treatment with standard weekly follow up at the same site without participating in study data collections. If a child presenting to a site assigned to monthly follow-up is eligible for SAM treatment but does not participate in the study (e.g. parent declines to provide consent or child does not meet inclusion criteria), the child will receive all medical and nutritional treatment with standard weekly follow up at the same site without participating in study data collections. Non-participating children seeking care at sites assigned to monthly follow-up will receive standard care, while all other eligible and participating children included at the monthly site will continue to receive the alternative monthly follow-up as per the random assignment of the site.

## 4.4 Informed consent

Following eligibility screening, a study nurse will introduce the study and ask if the parent/legal guardian would like to hear more about the study procedures. If the parent/legal guardian wishes to know more about the study, the research nurse will proceed with the consent process. A brief recruitment script is included in the Notice of Information.

The Notice of Information and Informed Consent Form will be read and discussed with all parents / legal guardians in their native language. If the parent / legal guardian is illiterate, a literate witness not connected to the research team will be present and sign and date the consent form to attest the consent process appears to be fair and the parent / guardian voluntarily agrees to participate. The parent / legal guardian will confirm voluntarily participation by signature or witnessed thumbprint.

A unique study identification number will be assigned to each participating child only after completion of the written informed consent procedures. An identification bracelet will be placed on the child and an identification card provided to the caregiver to facilitate identification of the child within the study at subsequent follow up and in the event of transfer to hospital. The identification card and bracelet will include the child’s study identification number but no other identifying information.

Note: informed consent preceding enrollment in the outpatient intervention study will be sought at all 10 outpatient therapeutic feeding centers. If a child is directly admitted to inpatient care (e.g. is not already consented and enrolled in the outpatient intervention study), separate informed consent will also be sought from at least one legal guardian or parent of children in order to specifically collect costing data related to inpatient treatment. When the hospitalized child is transferred from inpatient to outpatient care after stabilization, separate and full informed consent for enrollment in the outpatient intervention study will be sought at the time of outpatient admission.

## 4.5 Completion of admission visit

All caregivers providing consent will be interviewed with regard to socioeconomic/household characteristics and their knowledge, attitudes and practices related to surveillance of clinical danger signs.

At sites assigned to monthly follow up, a study nurse will also provide a short educational session to caregivers describing key danger signs for which the caregiver should return to the site for clinical evaluation, instruct caregivers how to monitor changes in MUAC at home, and introduce the take-home visual aids. Educational sessions may be provided individually or in small groups as needed, with the aim of reducing the wait time of caregivers while not diminishing the quality of training provided. The assessment of caregiver knowledge, attitudes and practices related to surveillance of clinical danger signs will be repeated after the educational session. Caregiver responses to the assessment conducted after the educational session will be reviewed with caregivers to insure correct comprehension of each danger sign for home surveillance. Caregivers will be reminded to return to the outpatient therapeutic feeding center if they believe their child’s condition worsens between scheduled study visits.

Note: children transferred from inpatient to outpatient care after stabilization will be eligible for enrollment at the time of outpatient admission. These children will follow the same enrollment activities described above, but follow-up will differ in that children treated at sites assigned to the monthly follow-up group will be asked to return at 1, 4, 8, 10 and 12 weeks until discharge.

At the end of the admission visit at all sites, the caregiver and child will receive all routine medicines and nutritional treatment sufficient until the next scheduled follow-up. RUTF provided to children enrolled in the study will be marked with a unique marking to facilitate identification of RUTF specifically provided by the study. At the end of the admission visit, compensation (e.g. 2 pieces of soap) for participation in the study will be provided to caregivers.

## 4.6 Follow-up visits

At sites assigned to standard weekly follow up, follow up visits at the study sites will be scheduled every week until discharge from the nutrition program for a maximum of 12 weeks. At sites assigned to the monthly follow up, follow up visits at the study sites will be scheduled at 4, 8, 10 and 12 weeks until discharge from the nutritional program. At all follow up visits, program staff will measure appetite, weight, height/length (discharge only), MUAC, edema and conduct a clinical examination.

The child will be referred to the inpatient center during follow-up for weight/edema changes (weight loss over 3 weeks; static weight over 4 weeks; onset of edema when previously absent; persistent edema 3 weeks following admission); failed appetite test; or clinical complication necessitating inpatient care (fever, hypothermia, severe dehydration, repeated vomiting, severe respiratory distress, chest in-drawing, severe pallor with respiratory distress, malaria with signs of severity, abscess or extended skin lesions, very weak, apathy or unconscious, or convulsions). A child admitted for inpatient care will be followed on a daily basis at the hospital while receiving inpatient treatment. After stabilization, the child will resume outpatient management and study follow-up at his/her assigned outpatient facility. If the child’s outpatient site was assigned to the monthly follow-up group, the child referred from inpatient care will be asked to return to the study site for outpatient management at 1, 4, 8, 10 and 12 weeks until discharge.

All caregivers will be instructed to return to the health center between scheduled visits if any clinical sign of concern develops at home. Spontaneous visits to a study health facility will be recorded with determination of the reason and treatment(s) provided.

At all follow-up visits, to understand utilization of the nutritional supplements in the household, all care givers will be asked to report average daily use of the supplement and return all finished and unfinished product.

At the discharge visit, caregivers knowledge, attitudes and practices related to the key danger signs and MUAC assessment for home-based surveillance will be assessed by standardized questionnaire (see Annex 9.2) in both the weekly and monthly follow-up groups. Compensation (e.g. 2 pieces of soap) for participation in the study will be provided to caregivers at the end of the discharge visit.

## 4.7 Unannounced safety visits within 2 weeks following inclusion

All children in both groups will receive an unannounced home visit by a study community health worker within the first two weeks of treatment. At each unannounced visit, the community health worker will directly observe RUTF stocks remaining at home, recording the number of sachets available in a household, and will assess the child’s MUAC, edema, and his/her clinical condition using a structured questionnaire (see Annex 9.2).

## 4.8 Tracing for missed visits (absences) and defaulting

We anticipate a non-negligible proportion of children will miss a scheduled visit and require tracing at home. The tracing of these children will be essential to insure complete data for a comparison of intervention groups.

To facilitate tracing of children for home visits, standard care includes notation of the child’s residence (ward and area) and caregiver’s name in the program registration book at admission. On a weekly basis at routine weekly meetings, program staff will inform community health worker volunteers of children who have missed scheduled visits in the previous week, and all children with a missed visit will be visited at home the following week. If the child is not present at the time of the home visit, relatives or neighbors will be contacted to collect information on vital status of the child if known.

As per standard care, and regardless of intervention assignment, all study children who have missed a scheduled visit will receive a home visit by a community health worker, who will determine vital status of the child and the reason for the missed visit. The community health worker will request the caregiver to return to the study site with the child as soon as possible for the scheduled medical and nutritional follow-up.

A child is considered as a “defaulter” after 3 consecutive missed visits in the weekly follow-up group, and after 1 missed visit in the monthly follow-up group. (e.g. maximum 3 weeks in the weekly follow-up group and maximum 4 weeks in the monthly follow-up group). No difference in the incidence/risk of complications is expected between the weekly and monthly follow-up groups due to the randomization. No difference in the detection of complications between groups is expected under the assumption that caregivers, with the educational session and take home materials provided, will be able to detect key danger signs at home in a manner similar to that observed at a health facility.

All defaulters will be visited at home by a study community health worker, who will determine vital status of the child and the reason for defaulting.

In the event of death, the Field Investigator will interview the family to obtain the cause of death using verbal autopsy methods and efforts will be made to retrieve medical records if the deceased was admitted to hospital.

## 4.9 Post-discharge follow-up

All children will receive a home visit by a community health worker 3 months following discharge from the nutritional program, regardless of type of discharge (e.g. recovered, default or non-recovered), requesting the caregiver and child to return to the outpatient therapeutic center. At each post-discharge visit, anthropometric measures (e.g. weight, height and MUAC) will be taken; history of nutritional treatment within the last 3 months will be assessed by maternal report; and caregivers knowledge, attitudes and practices related to the key danger signs and MUAC assessment will be assessed by standardized questionnaire (se Annex 9.2). Compensation (e.g. 2 pieces of soap) for participation in the post-discharge visit will be provided to caregivers at the end of the visit.

## 4.10 Standard care

Children will be treated according to the current national protocol for the management of SAM. Children are eligible for admission into the outpatient program if they are 6 to 59 months of age and fulfilled at least one of the following criteria: mid-upper arm circumference < 115 mm or grade 1-2 edema.

Children will be admitted directly to inpatient care if presenting with lack of appetite, intractable/continuous vomiting, fever, hypothermia, lower respiratory tract infection, severe respiratory distress, any chest in-drawing, severe anemia (hemoglobin < 7 g/dl), skin infection or lesions requiring IM/IV treatment, altered consciousness, hypoglycaemia, convulsions, severe dehydration, presence of grade 3 edema. Bipedal edema will be detected by the production of a pit after placing moderate pressure with the thumb on both legs over the top of the foot and lower end of the tibia for 3 seconds. All children, regardless of intervention assignment and as needed, may be subject to programmed home visits upon suspicion of social concerns, product sharing or clinician’s recommendation.

On admission, all children will receive amoxicillin; Vitamin A supplementation (if no edema and not already given within the last 1 month); de-worming; and measles vaccination (if not already given at 9 months). All children will be screened for plasmodium falciparum (malaria) using a rapid diagnostic test and treatment provided, if appropriate. Outpatient nutritional treatment will consist of RUTF, based on the weight of the child as per the national protocol.

## 4.11 Timeline

The total duration of proposed study is 36 months. The study timeline is summarized below.

1. Development and validation of education tools: 2014 (Niger)
2. Protocol and questionnaire development: February – March 2017
3. Ethical review: March 2017
4. Staff recruitment and training: April – May 2017
5. Adaptation of study tools to local context: April – May 2017
6. Pilot phase begins: June 2017
7. Study enrollment begins: March 2018
8. Enrollment complete: February 2019
9. Facility-based follow-up complete: April 2019
10. Home-based follow-up post discharge complete: July 2019
11. Data analysis and reporting: August – December 2019
12. Dissemination: January 2020

# 5.0 ETHICAL CONSIDERATIONS

## 5.1 Summary of known and potential risks

There is no large-scale data on the safety of a monthly schedule of follow-up in the treatment of uncomplicated SAM. However, based on the documentation of safety of outpatient treatment in other settings^8,9^ and preliminary results from a pilot study conducted in Niger which indicated that 1) caregivers satisfactorily retained information on clinical danger signs and the correct method to measure MUAC at home and 2) a monthly ration of RUTF can be safely distributed without a risk of weight loss or hospitalization, the risk associated with procedures in this study are expected to be minimal. All study procedures will be performed by adequately trained and experienced personnel under regular supervision to minimize any risk or discomfort to participants.

## 5.2 Risk minimization and benefits

Personnel involved providing clinical care to participants include Ministry of Health program staff and study staff. All such personnel are trained health care personnel and all (e.g. Ministry of Health program staff and study staff) will be provided additional training by the study team at the start of the study and regularly throughout the study duration. The additional training provided by the study team to health care personnel and study staff will refresh best practices and procedures to improve detection of clinical complications and will minimize risk associated with complications during treatment.

In addition, all caregivers will be instructed to return to the health center between scheduled visits if any clinical sign of concern develops at home. Encouragement for caregivers to return to the health center will promote timely management of any complication and will minimize risks associated with complications during treatment.

Caregivers assigned to a health center with a monthly schedule of follow-up will further receive additional training (30 minutes at the admission visit) and take-home pictorial tools for reference on how to assess clinical danger signs at home. This training specifically includes a presentation of the 9 clinical dangers signs for which care should be sought (e.g. diarrhea, vomiting, fever, lethargy, lack of appetite, edema, cough, difficulty breathing convulsions), as well as instruction on the procedure to measure the child’s anthropometric status at home using a modified MUAC tape. The recommended home surveillance of these 9 clinical danger signs and anthropometric assessment is equivalent to the surveillance of key clinical danger signs and anthropometric assessment conducted by health care personnel at the health facility during routine care. Caregivers’ knowledge of these signs and procedures will be assessed after training to insure complete and accurate understanding before continuing at home. Under the assumption that the training and take-home materials will allow caregivers to monitor children for key danger signs in an equivalent manner to that observed in a health facility, we expect no increased risk to children in the monthly follow-up group associated with monthly schedule.

The direct benefit individual participants in both groups may expect from participating in this study is seeing a member of the study team and the assurance of a high quality medical care (above that provided in usual care, given the additional staffing and resources provided by the study). Children and caregivers in the monthly follow-up group will also directly benefit from the additional education sessions, and take-home materials provided, to aid in the clinical surveillance of key danger signs while at home. The training can directly benefit the study child and others in the household by improving the caregiver’s capacity and self-efficacy. At the population-level, an important benefit of obtaining data on a more flexible schedule of follow-up, if proven safe and effectiveness, is the new model potentially being implemented at scale and increasing access to treatment to more children in need in Nigeria and beyond.

## 5.3 Informed consent

Information about the study aims and procedures will be provided to community leaders in all study villages before recruitment begins. No child will be included as a research participant without documented consent from at least one parent/legal guardian, which must be obtained prior to any study-related procedures are performed. Enrollment into the study will be made ​​voluntarily only after written informed consent. Individual children for whom consent is not provided will not participate in study data collections and will be provided all medical and nutritional treatment with standard follow-up regardless of study assignment (e.g. regardless if the site has, and all other participating children at the site have, been randomized to monthly follow-up).

The informed consent process will give individuals all of the relevant information they need to decide whether to participate and review two documents: a Notice of Information and an Informed Consent Form, both translated into the local language (see Annex 9.3). The informed consent process will begin with a study nurse describing the purpose of the study, procedures and potential risk and benefits of participation to the parent/guardian using the standardized Notice of Information. The study nurse will give the parent/guardian ample opportunity to inquire about details of the study and ask any questions. Illiterate individuals will have the Notice of Information and Informed Consent Form read to them in their native language in the presence of a literate and impartial witness. The witness will sign and date the consent form to attest the consent process appears to be fair and the parent/guardian voluntarily allows the child to be included. The study nurse who administered the consent procedure will sign to confirm that the consent has been obtained following the procedure described in the study protocol. A child will not be enrolled in the study until written informed consent for participation is provided.

One copy of the Informed Consent Form will be kept on file by the Field Investigator for possible inspection by representatives of the ethical committees. The participant’s parent/guardian will receive the Notice of Information and a second copy of the signed and dated Informed Consent Form.

Note: informed consent preceding enrollment in the outpatient intervention study will be sought at all 10 outpatient therapeutic feeding centers. If a child is directly admitted to inpatient care (e.g. is not already consented and enrolled in the outpatient intervention study), separate informed consent will also be sought from at least one legal guardian or parent of children in order to specifically collect costing data related to inpatient treatment. When the hospitalized child is transferred from inpatient to outpatient care after stabilization, separate and full informed consent for enrollment in the outpatient intervention study will be sought at the time of outpatient admission.

## 5.4 Confidentiality

All data collections will be conducted on a one-on-one basis between trained research and program staff and caregivers. A designated waiting area will allow for privacy and separation from other participants during data collection.

Participants will be identified by a unique individual identification number that contains no personal identifiable information. All records that contain names or other personal identifiers, such as informed consent forms and forms used for clinical follow-up, will be stored separately under lock and key. The participant's caregiver will be informed that representatives of the ethical committees may inspect their medical records to verify the information collected, and that all personal information made available for inspection will be handled in the strictest confidence. Participants’ study information will not otherwise be released outside of the study without the written permission of the participant’s caregiver. Personal identifiers will not be included in any study report.

All study records and data will be kept confidentially under lock and key and/or electronic password protection, as appropriate and in accordance with local data protection laws for 7 years. At the end of the 7 year storage period, the data will be destroyed in accordance with local data protection laws.

## 5.5 Reimbursement

There are no plans to provide monetary payment for participation in this study. However, as the study will require caregivers to visit a study facility for scheduled visits, the study will provide in-kind compensation for the additional burden associated with study data collection during the admission and discharge visits. The in-kind motivation for participation will include 2 pieces of soap distributed upon completion of the admission, discharge and post-discharge visits.

## 5.6 Research-related injuries

We do not anticipate any injuries to be associated with monthly vs. weekly follow-up beyond the background risks associated with SAM. If any child suffers any injury that is a result of his/her participation in this research, he/she will be treated at the nearest health center or hospital according to the current standard of care. If the injury is associated with participation in the study (e.g. beyond the background risks associated with SAM), the research will bear the cost of this treatment. There will be no other compensation outside of this medical treatment if a child is injured as a result of the study.

## 5.7 Storage of specimens

The study will not collect any biological specimens.

## 5.8 Institutional Review Board approval

The study will be approved by the research ethics committee of Harvard T.H. Chan School of Public Health and the National Health Research Ethics Committee, Nigeria and will be conducted in accordance with the Declaration of Helsinki.

## 5.9 Reportable events

The Field Investigator will report all adverse events or unanticipated problems involving risks to participants to the PI within 24 hours by email and telephone. The PI will report all adverse events or unanticipated problems involving risks to participants or others to the IRB within 5 business days of becoming aware of the event.

## 5.10 Declaration of conflict of interests

The Principal Investigator and co-investigator declare no conflict of interests.

## 5.11 Trial registration

The study will be registered with the US National Institutes of Health registry of clinical trials ([www.clinicaltrials.gov](http://www.clinicaltrials.gov)).

## 5.12 Regulatory compliance

The study will maintain a regulatory binder and use the Quality Improvement Program’s Investigator Self-Assessment (<https://www.hsph.harvard.edu/ohra/qip/study-management-tools>) once per year to ensure proper record keeping and retention of required regulatory documents.

# 6.0 PARTNERSHIPS

This study will be conducted by the Harvard School of Public Health, in collaboration with Epicentre, UNICEF Nigeria Country Office and the Nigerian Federal and State Ministry of Health. Médecins Sans Frontières-France will serve as a technical advisor.

Harvard School of Public Health will be responsible for the development of the protocol, supervision of study procedures, data analysis, and writing of the report for dissemination to all partners. The Principal Investigator will be responsible for the development of the protocol and will oversee study implementation. He/she will be in regular communication with field personnel regarding study coordination. An Epicentre Field Investigator will be based at the program site and be responsible for supervising the daily logistics and data collection activities of all study sites in compliance with the study protocol and standard operating procedures. The Field Investigator will be in routine communication with the Principal Investigator to provide regular updates on study progress (e.g. number on inclusions and follow up visits, number of follow up visits missed, storage of necessary supplies, data entry progress), ask questions when difficulties arise to avoid deviations from the protocol and to inform when a breach in protocol has occurred. All such study progress will also be provided in writing to the Principal Investigator in a weekly report.

Epicentre will be responsible for supervising outpatient and community-based follow-up and will support study administration in the field. UNICEF will ensure adequate RUTF supply to the study sites.

Investigators from all partners will be fully responsible for the ethical and scientific integrity of the proposed project. The Principal Investigator will regularly communicate with all partners to ensure all are informed of the study procedures and implementation.

# 7.0 ADMINISTRATIVE AND LOGISTICAL CONSIDERATIONS

## 7.1 Human resources

Each site will have a study team comprised of 2 study nurses, who will help take informed consent, collect background information at admission, and support high quality data collection at all enrollment and follow up visits, and 1 nutritional assistant who will support anthropometric assessment and data collection at all enrollment and follow up visits. There will be an additional study nurse assigned to the inpatient center, as well as 2 nurse supervisors to oversee activities at 5 sites each. Community health workers will conduct tracing of patients who do not attend regularly scheduled follow up and complete the unannounced safety visits, and issue reminders for the post-discharge follow-up visits. Four data entry clerks, under the supervision of a data manager, will conduct continuous double-data entry, comparison and cleaning. Members of the study team will be recruited locally for the duration of the study (approximately 19 months).

All locally recruited staff will be under the direct supervision of the Field Investigator, a trained medical doctor with experience in epidemiological research. The Field Investigator, recruited by Epicentre, will be responsible for the training and supervision of study staff and for coordination of all logistics and data collection activities in accordance with the study protocol and standard operating procedures.

## 7.2 Logistics

Substantial logistical considerations for study implementation include the following:

- Standard set-up of therapeutic feeding program, including adequate medical and nutritional supplies for the inpatient and outpatient treatment of SAM
- Adequate space within or near to the outpatient therapeutic feeding center for informed consent and collection of background information
- Adequate space within outpatient therapeutic feeding center for secure (e.g. locked) storage of data collection forms
- Adequate space within outpatient therapeutic feeding center for secure storage of study materials and treatments
- Clear channels for referral between study and program personnel, and between outpatient and inpatient services
- Office space for study staff
- Vehicles for study supervision
- Adequate security to allow long-term presence and movement of expatriate staff
- Operational network of community volunteers integrated into local CMAM program
- Availability of laptops for the Field Investigator and data entry team
- Appropriate compensation to be provided to caregivers at admission and discharge

# 8.0 REFERENCES

1. Black RE, Victora CG, Walker SP, et al. Maternal and child undernutrition and overweight in low-income and middle-income countries. Lancet 2013;382:427-51.

2. World Health Organization. Management of severe malnutrition: a manual for physicians and other senior health workers. Geneva: World Health Organization; 1999.

3. World Health Organization, World Food Program, United Nations System Standing Comittee on Nutrition, United Nations Children's Fund. Community-based management of severe acute malnutrition. Geneva: World Health Organization, the World Food Program, the United Nations System Standing Comittee on Nutrition, and the United Nations Children's Fund; 2007.

4. Khara T, Collins S, eds. Community-based Therapeutic Care (CTC) Special Supplement. Oxford: Emergency Nutrition Network; 2004.

5. UNICEF, Coverage Monitoring Network, ACF International. The State of Global SAM Management Coverage 2012 New York & London: UNICEF, Coverage Monitoring Network and ACF International; 2012.

6. National Bureau of Statistics Nigeria. National Nutrition and Health Survey 2015: Report on the nutrition and health situation of Nigeria. Abuja: National Bureau of Statistics; 2015.

7. Farrington CP, Manning G. Test statistics and sample size formulae for comparative binomial trials with null hypothesis of non-zero risk difference or non-unity relative risk. Statistics in medicine 1990;9:1447-54.

8. Ciliberto MA, Sandige H, Ndekha MJ, et al. Comparison of home-based therapy with ready-to-use therapeutic food with standard therapy in the treatment of malnourished Malawian children: a controlled, clinical effectiveness trial. The American journal of clinical nutrition 2005;81:864-70.

9. Manary MJ, Ndkeha MJ, Ashorn P, Maleta K, Briend A. Home based therapy for severe malnutrition with ready-to-use food. Archives of disease in childhood 2004;89:557-61.

# 9.0 ANNEXES

9.1 Sample educational tools for caregiver training on home-based surveillance of clinical danger signs and MUAC assessment

9.2 Draft case report forms

9.3 Information Sheet and Informed Consent Form
