# Supplementary material for: Effectiveness of a monthly schedule of follow-up for the treatment of uncomplicated severe acute malnutrition in Sokoto, Nigeria: A cluster randomized crossover trial
Source: PLoS Med. 2022 Mar 1;19(3):e1003923. doi: 10.1371/journal.pmed.1003923 (PMC8887725; doi:10.1371/journal.pmed.1003923)
Supplement: S1 Text — (DOCX) [file pmed.1003923.s003.docx]

Effectiveness of a monthly schedule of follow-up for the treatment of uncomplicated severe acute malnutrition in Sokoto, Nigeria: a cluster-randomized crossover trial

**Supplementary Tables**

Matt Hitchings^1,2^, Fatou Berthé^3^, Philip Aruna^4^, Ibrahim Shehu^3^, Muhammed Ali Hamza^5^, Siméon Nanama^6^, Chizoba Steve-Edemba^7^, Rebecca F. Grais^8^, and Sheila Isanaka^8,9^

Table A. Effect of monthly schedule of follow-up compared to standard weekly schedule of follow-up on outcomes assessed at program discharge, by season

|  | % outcome in lean season (weekly/ monthly) | Risk ratio in lean season (95% CI) | % outcome out of lean season (weekly/ monthly) | Risk ratio out of lean season (95% CI) | P value for interaction |
| --- | --- | --- | --- | --- | --- |
| N (weekly/monthly) |  | 958/994 |  | 844/982 | **-** |
| Nutritional recovery | 61.3%/ 51.9% | 0.84 (0.75,0.93) | 55.9%/ 53.0% | 0.94 (0.84,1.05) | 0.05 |
| Non-response | 25.3%/ 36.5% | 1.44 (1.17,1.77) | 33.2%/ 37.1% | 1.14 (0.95,1.38) | 0.01 |
| Death | 4.5%/6.0% | 1.39 (0.97,1.99) | 3.2%/4.5% | 1.45 (0.93,2.28) | 0.88 |
| Default | 9.0%/5.5% | 0.67 (0.33,1.34) | 7.7%/5.5% | 0.68 (0.34,1.37) | 0.92 |
| Hospitalization | 18.1%/ 15.5% | 0.86 (0.65,1.13) | 12.3%/ 11.5% | 0.92 (0.66,1.30) | 0.64 |
| Due to weight loss or oedema | 2.8%/8.6% | 2.98 (1.90,4.69) | 3.0%/6.7% | 2.22 (1.38,3.57) | 0.35 |
| Due to clinical complications | 14.5%/8.5% | 0.58 (0.44,0.77) | 10.1%/ 6.7% | 0.65 (0.47,0.91) | 0.54 |
| Hospitalization or death | 21.4%/ 20.5% | 0.97 (0.77,1.22) | 14.9%/ 15.3% | 1.01 (0.76,1.35) | 0.74 |

Table B. Causes of death during treatment, by group

| Cause of death | Overall (n=172*) | Weekly group (n=68) | Monthly group (n=104) |
| --- | --- | --- | --- |
| Cerebral malaria | 5 (2.9%) | 3 (4.4%) | 2 (1.9%) |
| Choking | 1 (0.6%) | 1 (1.5%) | 0 (0%) |
| Gastroenteritis | 84 (48.8%) | 30 (44.1%) | 54 (51.9%) |
| Gastrointestinal disorder | 1 (0.6%) | 1 (1.5%) | 0 (0%) |
| Kwashiorkor | 3 (1.7%) | 1 (1.5%) | 2 (1.9%) |
| Lower respiratory tract infection | 31 (18.0%) | 13 (19.1%) | 18 (17.3%) |
| Malaria | 30 (17.4%) | 11 (16.2%) | 19 (18.3%) |
| Marasmus | 7 (4.1%) | 3 (4.4%) | 4 (3.9%) |
| Measles | 1 (0.6%) | 1 (1.5%) | 0 (0%) |
| Meningitis | 1 (0.6%) | 0 (0%) | 1 (1.0%) |
| Post procedural infection | 1 (0.6%) | 1 (1.5%) | 0 (0%) |
| Sickle cell anaemia | 1 (0.6%) | 0 (0%) | 1 (1.0%) |
| Staphylococcal skin infection | 1 (0.6%) | 1 (1.5%) | 0 (0%) |
| Thermal burn | 1 (0.6%) | 0 (0%) | 1 (1.0%) |
| Toxicity to various agents | 1 (0.6%) | 1 (1.5%) | 0 (0%) |
| Unspecified relapsing fever | 3 (1.7%) | 1 (1.5%) | 2 (1.9%) |

* Two children did not have a cause of death reported
